# MiRNAs as potential therapeutic targets and biomarkers for non-traumatic intracerebral hemorrhage

**DOI:** 10.1186/s40364-024-00568-y

**Published:** 2024-02-02

**Authors:** Ilgiz Gareev, Ozal Beylerli, Boxian Zhao

**Affiliations:** 1 Department of Neurosurgery, The First Affiliated Hospital of Harbin Medical University, No. 23 Youzheng Street, Nangang District, Harbin, 150001 China; 2 Bashkir State Medical University, Ufa, 450008 Russia; 3 Harbin Medical University No, 157, Baojian Road, Nangang District, Harbin, 150001 China

**Keywords:** Non-traumatic intracerebral hemorrhage, miRNA, Circulating miRNAs, Biomarker, Therapy, Preventive, Diagnosis, Prognosis, Theory

## Abstract

Non-traumatic intracerebral hemorrhage (ICH) is the most common type of hemorrhagic stroke, most often occurring between the ages of 45 and 60. Hypertension is most often the cause of ICH. Less often, atherosclerosis, blood diseases, inflammatory changes in cerebral vessels, intoxication, vitamin deficiencies, and other reasons cause hemorrhages. Cerebral hemorrhage can occur by diapedesis or as a result of a ruptured vessel. This very dangerous disease is difficult to treat, requires surgery and can lead to disability or death. MicroRNAs (miRNAs) are a class of non-coding RNAs (about 18-22 nucleotides) that are involved in a variety of biological processes including cell differentiation, proliferation, apoptosis, etc., through gene repression. A growing number of studies have demonstrated miRNAs deregulation in various cardiovascular diseases, including ICH. In addition, given that computed tomography (CT) and/or magnetic resonance imaging (MRI) are either not available or do not show clear signs of possible vessel rupture, accurate and reliable analysis of circulating miRNAs in biological fluids can help in early diagnosis for prevention of ICH and prognosis patient outcome after hemorrhage. In this review, we highlight the up-to-date findings on the deregulated miRNAs in ICH, and the potential use of miRNAs in clinical settings, such as therapeutic targets and non-invasive diagnostic/prognostic biomarker tools.

## Introduction

**Fig. 1:**
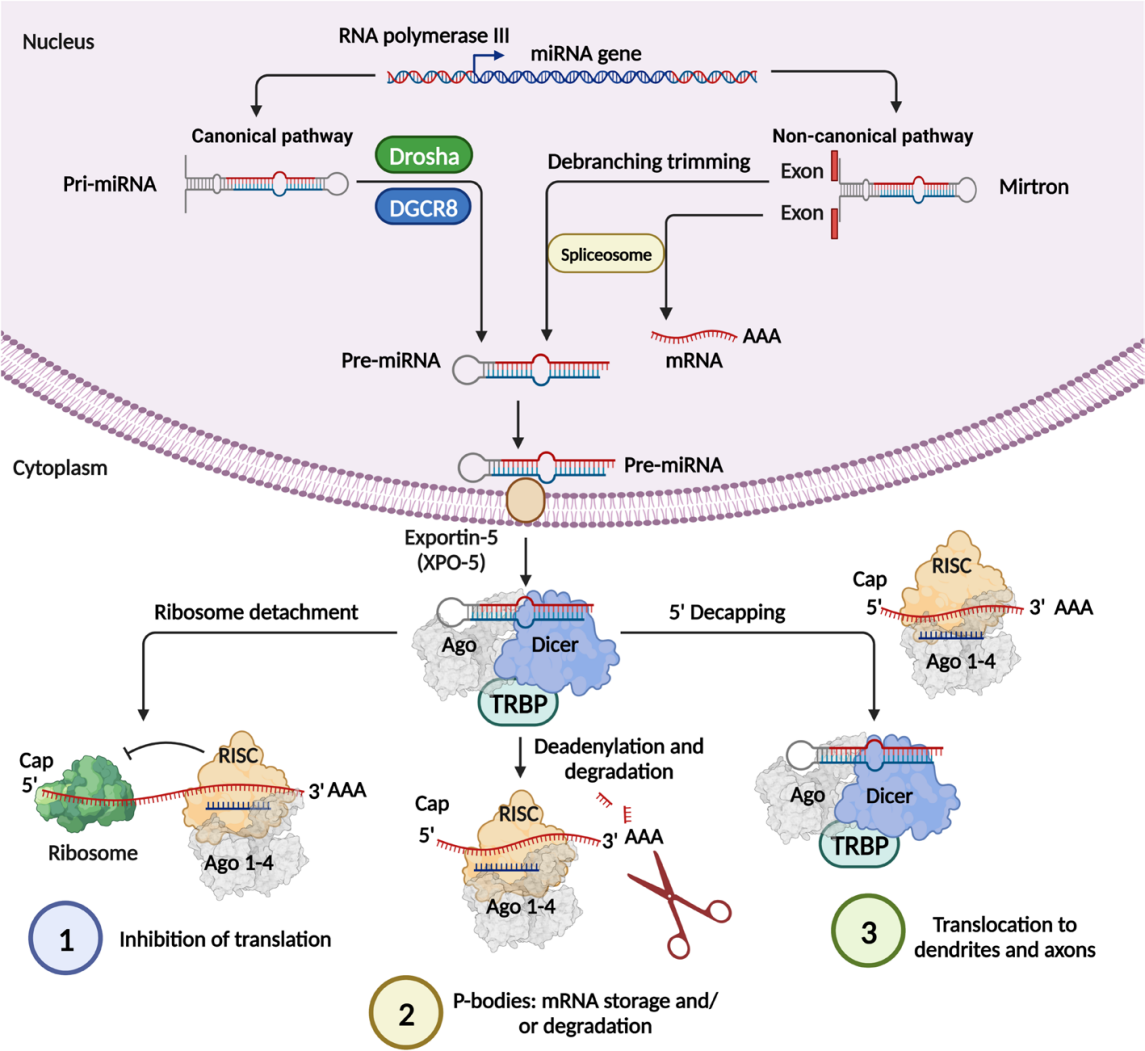
MicroRNA (miRNA) biogenesis. MiRNA biogenesis is well studied and described in detail. It is a multi-step process regulated by several enzymes where the formation of mature miRNA proceeds in two ways, canonical and non-canonical. Note: pri-miRNA, primary microRNA; pre-miRNA, precursor microRNA; RISC, RNA-induced silencing complex; mRNA, messenger RNA; Ago, Argounaute; TRBP, Transactivation response element RNA-binding protein

Non-traumatic intracerebral hemorrhage (ICH) is local bleeding into the parenchyma of the brain, which occurs because of rupture of cerebral blood vessels [1]. ICH usually develops when an atherosclerotic cerebral artery ruptures, the wall of which has undergone changes because of a prolonged existing increase in blood pressure [1]. The problem of early diagnosis, prognosis and treatment of ICH is one of the most important in modern medicine. Despite the vast experience of modern neurosurgery and neurology in the treatment of patients with ICH, the tactics of managing patients are still controversial, and indications for various methods of therapy need to be clarified [2]. Until now, neither conservative nor surgical methods of treatment have a clear advantage. Therefore, the study of the molecular mechanisms of the pathogenesis of ICH will deepen the understanding of the course of ICH and clarify some issues of diagnosis and treatment tactics. The mechanisms of ICH development include impaired blood-brain barrier (BBB) function and cerebral edema, cell apoptosis, inflammation, oxidative stress, activation of signaling pathways that regulate angiogenesis, endothelial disfunction, and suppression of signaling pathways responsible for maintaining the phenotype of vascular smooth muscle cells (VSMCs) [3]. MicroRNAs (miRNAs) are short, on average 18-22 nucleotides, single-stranded non-coding RNAs that post-transcriptional regulate gene expression by binding to the 3'-untranslated region (3'-UTR) of the messenger RNA (mRNA) target, which ultimately leads to decreasing protein expression by blocking translation and/or promoting degradation of the target mRNA [3] (Fig. 1). Many studies have shown that miRNAs play an important regulatory role in the occurrence and development of cerebrovascular diseases (CVDs). The mechanisms by which miRNAs play a role in the development of CVD are very complex (Table 1) [4–18]. The result of the interaction between miRNA and mRNA depends on the degree of complementarity. Full or partial complementarity between the key sequence (seed sequence) of the miRNA, located from the 1st or 2nd to the 7th or 8th nucleotide at the 5’ end, and the mRNA region ensures landing on the target mRNA. With complete complementarity, the RNase activity of Argonaute 2 (Ago2) is activated, which cuts the mRNA at the landing site, the latter is cleaved by ribonucleases and degraded. When the interaction of the seed region with the target is incomplete (the seed region is too short, forms a loop, or contains non-complementary nucleotides), the miRNA exhibits its function if some of its nucleotides at positions 12–17 also bind to the target. Then it is not mRNA cleavage that occurs, but translation suppression [19, 20].

**Table 1:** Results of studies on cerebrovascular microRNAs (miRNAs)

**Neurovascular unit**	**MiRNA**	**Targets**	**Important find**	**References**
OPCs	MiR-3074-3p	Cav-1	Great clinical value in ischemic demyelination. Ameliorates OPCs clustering and promotes oligodendrogenesis	[4]
Microglia	MiR-195	CX3CL1/CX3CR1 signaling pathway	Regulates of microglia M1 polarization	[5]
OPCs	MiR-23a-5p	Olig3	Promotes white matter remodeling and repair in stroke and demyelinating diseases	[6]
ECs	MiR‐140‐5p	VEGFA	Promotes proliferation, migration, and tube formation	[7]
ECs	Mir‐126‐3p and miR‐126‐5p	IL‐1β, TNF‐α, VCAM‐1, E‐selectin	Maintenances of BBB integrity	[8]
ECs	MiR‐107	Dicer‐1	Promotes angiogenesis	[9–11]
	MiR‐24‐1‐5p	HIF‐1α		
	MiR‐191	NF‐kβ		
VSMCs	MiR-552	ATF4 and SKI	Critical role in the proliferation and migration	[12]
VSMCs	MiR-137	Calcineurin/NFATC3 signaling pathway	Modulates the dedifferentiation and proliferation	[13]
Pericytes	Let-7d	bFGF	Induces of pericyte differentiation	[14]
Astrocytes	MiR-182-5p	IL-1β, IL-6 and TNF-α	Inhibits neuroinflammation and improves brain injury after ischemic stroke	[15]
Neurons	MiR‐106b‐5p	Mcl‐1/Bcl‐2 signaling pathway	Inhibits of apoptosis and oxidative stress	[16]
Neurons	MiR‐149‐5p	P53/caspase‐3 signaling pathway	Regulates of cell survival and apoptosis	[17]
Neurons and astrocytes	MiR‐19a‐3p	ADIPOR2	Modulates of glucose metabolism and neuronal apoptosis	[18]

*Abbreviations*: *EC* Endothelial cells, *VSMCs* Vascular smooth muscle cells, *OPCs* Oligodendrocyte precursor cells, *BBB* Blood–brain barrier, *Cav-1* Caveolin-1, *CX3CL1* Chemokine (C-X3-C motif) ligand 1, *CX3CR1* CX3C motif chemokine receptor 1, *Olig3* Oligodendrocyte transcription factor 3, *VEGFA* Vascular endothelial growth factor A, *IL‐1β* Interleukin 1β, *TNF‐α* Tumor necrosis factor-alpha, *VCAM‐1* Vascular cell adhesion molecule 1, *HIF‐1α* Hypoxia-induced factor-1alpha, *NF‐kβ* Nuclear factor kappa β, ATF4, Activating transcription factor 4, *SKI* Proto-oncogene *bFGF* Basic fibroblast growth factor, *NFATC3* Nuclear factor of activated T-cells, *IL-6* Interleukin 6, *Mcl‐1* Myeloid leukemia 1, *Bcl‐2* B-cell lymphoma 2, *P53* Tumor protein p53, *ADIPOR2* Polyclonal antibody to adiponectin receptor 2

MiRNAs can negatively modulate the activity of target genes and, therefore, regulate protein synthesis, affecting the stability of the mRNAs encoding them. In addition, miRNAs can also regulate functionally related proteins and have specific effects on the formation of protein complexes and biological pathways [21]. Therefore, to understand the function of miRNAs and their role more clearly in diseases, including ICH, research on miRNA biology must be conducted in the context of a protein interaction network rather than isolated target genes. Moreover, proteins mediated by the same miRNA have a high propensity to interact with each other. These specific characteristics imply that miRNAs may exert their regulatory effects on protein complexes and pathways through a network of protein interactions [22]. Thus, based on the analysis of the network of protein interactions mediated by miRNAs, it is not possible to fully understand the function of miRNAs, but to more accurately identify miRNAs associated with ICH.

In addition, miRNA expression is controlled through DNA modification, such as methylation and transcription factors through the signal transduction pathway [23]. DNA methylation refers to the formation of a covalent bond between the 5' cytosine carbon of a DNA CpG dinucleotide (5'-cytosine-guanine-3') and a methyl group by DNA methyltransferase, forming 5-methylcytosine [23]. DNA hypermethylation can directly inhibit transcription or indirectly inhibit gene expression through transcriptional repression [24]. Changes in DNA methylation is associated with CVDs such as ischemic stroke (IS) and ICH [25, 26]. The hypermethylation of the CpG-rich promoter regions of vascular-specific miRNAs usually leads to their silencing through DNA methyltransferase (DNA MTase, DNMT) [27]. The abnormal DNA methylation of miRNA usually leads to the downregulation of miRNA, which is significantly related to the phenotypic switching of VSMCs or endothelial cells (ECs) disfunction [26]. In turn, miRNA could regulate the expression level of the target gene after transcription, which affects DNA methylation in ECs and VSMCs through the 3′-UTR of the RNA-induced silencing complex (RISC)-targeted DNMT mRNA [27]. In conclusion, this demonstrates the existence of a regulatory loop between miRNA expression and epigenetic modifications.

In recent years, the attention of researchers has been attracted by the study of changes in the expression levels of circulating miRNAs in various biological fluids of the human body for their potential consideration as non-invasive diagnostic and prognostic biomarkers of vascular dysfunction or damage brain tissue in CVDs (Table 2) [28–36]. Importantly, the specific expression signatures of circulating miRNAs in biological fluids reflect not only the existence of early-stage diseases, but also the dynamic development of late-stage diseases, disease prognosis, and treatment monitoring [19]. To date, a growing number of circulating miRNAs have been reported as potential noninvasive biomarkers in ICH, but the prospects for their practical application are still unclear. Thus, a summary of the circulating miRNA expression profile is of great importance for the identification of new promising biomarkers in ICH.

**Table 2:** Overview of circulating microRNAs (miRNAs) in patient’s biological fluids with cerebrovascular diseases. Note: studies examining non-traumatic intracerebral hemorrhage (ICH) were not included

**Disease**	**MiRNA**	**Regulation**	**Biological fluid**	**Important finding**	**References**
AIS	MiR-451a	Down	Serum	Diagnostic significance. Significantly associated with poor clinical outcomes of stroke, as defined by the modified Rankin scores	[28]
AIS	EV-derived miR-19a-3p, miR-186-5p and let-7f	Up	Plasma	Diagnostic significance. Combining circulating miRNAs might be beneficial for early diagnosis of ischemic events	[29]
AIS	Exosomal miR-223	Up	Serum	Diagnostic significance. Associated with acute ischemic stroke occurrence, stroke severity, and short-term outcomes	[30]
AIS and TIA	MiR-195-5p and miR-451	Up and down	Serum	At admission were significantly upregulated and significantly decreased over time at 24 h and 48 h, and it is associated with decreased HIF-α levels and increased VEGF serum levels	[31]
aSAH	Exosomal miR-369-3p, miR-193b-3p, and miR-486-3p	Up	Plasma	Diagnostic significance. Significantly associated with poor clinical outcomes of aSAH, as defined by the WFNS grade, Hunt–Hess grade, and Fisher score	[32]
aSAH	MiR-132 and miR-324	Up	Plasma	Diagnostic significance. Correlation with DCI	[33]
aSAH and UIA	MiR-126	Up	Plasma	Potential non-invasive biomarker in UIAs detection and prevention aneurysms with a high risk of rupture	[34]
aSAH and UIA	MiR-183-5p and let7b-5p	Down	Plasma	Potential non-invasive biomarkers in UIAs detection and prevention aneurysms with a high risk of rupture	[35]
	MiR-200a-3p	Up			
BAVM	MiR-7-5p, miR-199a-5p and miR-200b-3p	Up	Whole blood	Diagnostic significance	[36]

*Abbreviations*: *AIS* Acute ischemic stroke, *TIA* Transient ischemic attack, *aSAH* Aneurysmal subarachnoid hemorrhage, *UIA* Unruptured intracranial aneurysm, *BAVM* Brain arteriovenous malformation, *DCI* Delayed cerebral infarction, *HIF-α* Hypoxia-inducible factor α, *VEGF* Vascular endothelial growth factor, *WFNS* World Federation of Neurosurgeons

This review summarizes the brief data on miRNAs that have been proven to be involved in the pathogenesis of ICH, their targets, and mechanisms of action, and summarizes the latest advances in the context of the potential application of miRNAs in clinical practice, particularly, the possibility of their use in therapy and for early diagnosis, prognosis, and therapy monitoring for ICH. In addition, we will discuss important general questions about the possibilities of using as therapy drugs and biomarkers in ICH, uncovering the benefits of circulating miRNAs.

## MiRNAs and the main pathogenetic elements of ICH

ICH is a multifactorial disease with many established causes. A key role in the pathogenesis of ICH is played by endothelial dysfunction and pronounced changes in the walls of cerebral vessels under the influence of chronically elevated blood pressure and atherosclerosis [1, 2]. The molecular basis for the development of ICH is complex and includes a whole range of disorders, among which the apoptosis of ECs of cerebral vessels and neurons, the inflammatory process and the response of the immune system, oxidative stress, impaired BBB function, and cerebral edema occupy a central place [1, 2]. It is widely known that miRNAs are powerful regulators involved in all molecular and cellular processes, both in normal conditions and in various diseases [21, 22]. Understanding the regulation of miRNAs and their targets makes it possible to determine the causes of the occurrence of ICH caused by changes in gene expression. Several *in vitro* and *in vivo* studies have provided clear evidence of the involvement of miRNAs in the pathogenesis of the development and progression of ICH, which will be described in this chapter (Table 3) [37–73].

**Table 3:** MicroRNAs (miRNAs) and their target genes involved in the main elements of pathogenesis non-traumatic intracerebral hemorrhage (ICH). Note: *in vitro* and *in vivo* studies, expression level shown during application anti-miRNA or miRNA mimics

**MiRNA**	**Expression**	**Process**	**Study model**	**Targets**	**Mechanism**	**References**
MiR-126	Up	Apoptosis	C57BL/6 mice and BMECs	VEGF-A	Inhibits of ECs apoptosis after ICH	[37]
MiR-126	Up	Apoptosis	SD rats and rats brain tissue	VEGF-A and caspase-3	May significantly improve behavioral performance in ICH. Decrease the damage to the brain and cell apoptosis	[38]
MiR-126	Up	Apoptosis and neuroinflammation	SD rats and rats brain tissue	ZEB1, GFAP, TNF-α, IL-1β, and IL-6	Anti-inflammatory effect and inhibits of neuronal apoptosis in hematoma area	[39]
MiR-126-3p	Up	BBB permeability, cerebral edema, apoptosis, and neuroinflammation	SD rats and BMECs	PIK3R2/Akt axis	Reduces brain edema and BBB permeability. Inhibits neutrophil infiltration, microglial activation, and neuronal apoptosis	[40]
MiR-130a	Down	BBB permeability, and cerebral edema	SD rats and rats BMECs	Cav-1/MMP-2/MMP-9 signaling pathway	Attenuates brain edema, preserves BBB integrity and improves neurological behavioral functions score	[41]
MiR-132	Up	BBB permeability, cerebral edema, apoptosis, and neuroinflammation	C57BL/6J mice and mouse brain tissue	AChE, IL-6, IL‐1β and TNF-a	Protective effect on BBB permeability, anti-inflammatory effect and inhibits of neuronal apoptosis in hematoma area	[42]
MiR-27a-3p	Up	BBB permeability, cerebral edema, apoptosis, and neuroinflammation	SD rats and rats BMECs	AQP-11	Reduces neuronal apoptosis, brain edema, BBB permeability and microglia activation in the perihematomal area	[43]
MiR-126-3p	Up	BBB permeability	SD rats and rats BMECs	VCAM-1	Reduces BBB permeability	[44]
MiR‐21‐5p	Down	BBB permeability, neuroinflammation and apoptosis	Sprague-Dawley rats and HEK293T cells	HO‐1, DUSP8, IL‐1β, IL‐6 and TNF‐α	Reduces the neurological defects and BBB permeability, repairs cognitive impairment, permeability, anti-inflammatory effect and inhibits neuronal apoptosis in the perihematomal area	[45]
MiR-126-3p	Up	BBB permeability and apoptosis	SD rats and rats brain tissue	ZO-1, claudin-5, RAR-1, and MMP-9	Reduces BBB permeability and inhibits neuronal apoptosis in the perihematomal area	[46]
MiR-18a	Up	BBB permeability and apoptosis	SD rats, rats BMECs, and rats astrocytes	RUNX1-Occludin/ZO-1 axis, MMP-2, MMP-9 and TIMP-1	Reduces BBB permeability and inhibits neuronal apoptosis in the perihematomal area.	[47]
MiR-137	Up	Oxidative stress and apoptosis	SH-SY5Y cells and human EPCs	COX2/PGE2 pathway	Neuroprotective effect	[48]
MiR-29a	Up	Oxidative stress and apoptosis	SD and culture neurons	PTEN/PI3K/Akt/mTOR signaling pathway	Promotes regenerative outgrowth of injured axons and improves neurobehavioral and cognitive impairments. Alleviates neuronal damage and mitochondrial dysfunctions, and facilitates neurite outgrowth	[49]
MiR-146a-5p	Up	BBB permeability, oxidative stress, apoptosis, and neuroinflammation	SD rats and rats brain tissue	iNOS, COX-2, MCP-1, IRAK-1, and NFAT-5	Reduces neuronal apoptosis and inflammation (inhibition of microglial M1 polarization)	[50]
MiR-183-5p	Up	Oxidative stress, apoptosis and neuroinflammation	C57BL/6 mice, and BV2 microglial cell line	HO-1 and Nrf2.	Reduces BBB permeability, inflammation, ROS production and inhibits neuronal apoptosis in the perihematomal area	[51]
MiR-331-3p	Down	Neuroinflammation	C57BL/6 mice, and BV2 microglial cell line	NLRP6, TNF-α and IL-6.	Alleviates the inflammatory response and promotes the neurological function recovery	[52]
MiR-378a-5p	Down	BBB permeability, oxidative stress, apoptosis, and neuroinflammation	C57BL/6J mice and mouse brain tissue	Hspa5, IL-1β, IL-6, and TNF-α	Reduces BBB permeability, Alleviates the inflammatory response and promotes the neurological function recovery	[53]
MiR-34a-5p	Up	Apoptosis and neuroinflammation	SD rats and rats brain tissue	KLF-4	Inhibits microglia M2 polarization while accelerating M1 polarization and anti-apoptotic effects in the perihemorrhagic penumbra	[54]
MiR-383-3p	Up	Neuroinflammation	SD rats and BV2 microglial cell line	ATF4	Activates microglia and promotes neuronal necroptosis	[55]
MiR-150-3p	Up	Gut microbiota, metabolism, apoptosis, and neuroinflammation	C57BL/6J mice and mouse brain tissue	TRAF6/NF-κB axis	Reduces apoptosis and levels of inflammatory factors	[56]
MiR‑93‑5p	Down	Apoptosis and neuroinflammation	Thrombin‑treated human microglial HMO6 cells	TGF‑β1 and Nrf2	Reduces neuronal apoptosis and inflammation	[57]
MiR-124-3p	Up	Apoptosis and neuroinflammation	HMC3 cells	TRAF6	Reduces the inflammatory factors and apoptosis rate	[58]
MiR-23a-3p	Up	Apoptosis	hCMEC/D3 cells	ZO-1	Decreases vascular endothelial cells proliferation and increases their apoptosis	[59]
MiR-146a	Up	Oxidative stress, apoptosis and neuroinflammation	SD rats and hCMEC/D3	TRAF6/NF-κB signaling pathway, MDA, SOD, GSH-Px, TNF-α and IL-1β	Reduces BBB permeability, pro-inflammatory cytokine production, oxidative stress inhibits and neuronal apoptosis	[60]
MiR‑155	Down	Neuroinflammation	SD rats and astrocytes	SOCS‑1, IFN‑β, TNF‑α and IL‑6	Anti-inflammatory effect	[61]
MiR-27b	Down	Oxidative stress, apoptosis and neuroinflammation	SD rats and PC12 cells	Nrf2/ARE signaling pathway	Reduces the lipid peroxidation, neuroinflammation, cell death and neurological deficits	[62]
MiR-25-3p	Up	Oxidative stress, apoptosis and neuroinflammation	Human blood (plasma) and C57BL/6 mice	P53/SLC7A11/GPX4 signaling pathway	Neuroprotective role against ferroptosis. Promotes locomotion recovery of ICH mice.	[63]
MiR-133b	Up	Apoptosis	C57BL/6 mice and mouse brain tissue	FOXO4/Bcl-2 axis	Inhibits of hippocampal neuronal apoptosis	[64]
MiR-23b	Up	Oxidative stress, apoptosis and neuroinflammation	Wistar rat BMSCs, HT22 cells and Wistar rats	PTEN/Nrf2 signaling pathway	Improves behavioral functions and reduces brain injuries. Inhibits the amount of microglia/macrophages and cell apoptosis. Reduces oxidative stress	[65]
MiR-185-5p	Down	Apoptosis	C57BL/6J mice, 293T cell line and HT22 cell line	4933404O12Rik/Sh2b3/ERK1/2 signaling pathway	Anti-apoptotic effect	[66]
MiR-488	Up	Oxidative stress and apoptosis	C57BL/6J mice and mouse brain tissue	CLSTN3	Increases cell viability and reduced markers of oxidative stress and lipid peroxidation	[67]
MiR-29a-3p	Up	Apoptosis	C57BL/6J mice and HT22 cell line	SOX10/ACSL4 axis	Contributes to ferroptosis of hippocampal neurons after ICH and inhibits the iron death of hippocampal neuronal cells	[68]
MiR-124-3p	Down	Apoptosis	Human blood (plasma) and HMC3 cell	MTF1	Inhibits cell viability and enhances cell apoptosis	[69]
MiR-124	Up	BBB permeability, oxidative stress, apoptosis, and neuroinflammation	C57BL/6J mice and BV2 microglia	ZO-1, claudin-5, MMP-9, IL-1β, IL-6, and TNF-α	Attenuates BBB damage, reduces cell death and inhibits brain inflammation after ICH. Attenuates neurological deficits and brain edema. Reduces immune cell infiltration	[70]
MiR-106a-5p	Down	BBB permeability, oxidative stress, and apoptosis	C57BL/6J mice and mouse brain tissue	Nrf2/ARE signaling pathway and PHLPP2	Neuroprotective role.	[71]
MiR-122-5p	Down	BBB permeability, apoptosis, and neuroinflammation	C57BL/6J mice and BV2 microglia	MLLT1/PI3K/Akt signaling pathway	Decreases neurological deficits, BBB permeability, and inflammation	[72]
MiR-204-5p	Down	Neurological regeneration	SD rats and rats brain tissue	CLCN3	Inhibits neuronal injury, improves neurological function, and then alleviates ICH	[73]

*Abbreviations*: *ECs* Endothelial cells, *BBB* Blood-brain barrier, *SD rats* Sprague-Dawley rats, *BMECs* Brain microvascular endothelial cells, *EPCs* Endothelial progenitor cells, *SH-SY5Y cells*, Thrice-cloned sub-line of bone marrow biopsy-derived line SK-N-SH, *PIK3R2* Phosphatidylinositol 3-kinase regulatory subunit beta, *AChE* Acetylcholinesterase, *IL-6* Interleukin 6, *TNF-a* Tumor necrosis factor alpha, *AQP11* Aquaporin-11, *VCAM-1* Vascular cell adhesion molecule 1, *HO‐1* Oxygenase‐1, *DUSP8* Dual-specificity phosphatase 8, *IL‐1β* Interleukin 1 Beta, *ZO-1* Zonula occludens-1, *RAR-1* Protease activated receptor-1, *RUNX1* Runt-related transcription factor 1, *MMP-2* Matrix metalloproteinase 2, *MMP-9* Matrix metalloproteinase 9, *TIMP-1* Metallopeptidase inhibitor 1, *PGE2* Prostaglandin E2, *PTEN* Phosphatase and tensin homolog, *PI3K* Phosphoinositide 3-kinases, *mTOR* Mammalian target of rapamycin, *iNOS* Nitric oxide synthase, *COX-2* Cyclooxygenase-2, *MCP-1* Monocyte chemoattractant protein-1, *IRAK1* Interleukin-1 receptor-associated kinase1, *NFAT5* Nuclear factor of activated T cells 5, *Nrf2* Nuclear factor erythroid 2–related factor 2, *NLRP6* NOD-like receptor family pyrin domain containing 6, *Hspa5* Heat shock protein family A (Hsp70) member 5, *KLF-4* Krüppel-like factor 4, *ATF4* Activating transcription factor 4, *TRAF6* TNF receptor associated factor 6, *NF-κB* Nuclear factor kappa-light-chain-enhancer of activated B cells, *TGF‑β1* Transforming growth factor β1, *MDA* Malondialdehyde, *SOD* Superoxide dismutase, *GSH-Px* Glutathione peroxidase, *TNF-α* Tumour necrosis factor alpha, *SOCS‑1* Suppressor of cytokine signaling 1, *IFN‑β* Interferon beta, *ZEB1* Zinc finger E-box binding homeobox 1, *GFAP* Glial fibrillary acidic protein, *Cav-1* Caveolin-1, *P53* Tumor protein p53, *SLC7A11* Cystine/glutamate transporter, *GPX4* Glutathione peroxidase 4, *FOXO4* Forkhead box O4, *Bcl-2* B-cell leukemia/lymphoma 2, *4933404O12Rik* RIKEN cDNA 4933404O12, *Sh2b3* SH2B adaptor protein 3, *ERK1/2* Extracellular signal-regulated kinase 1/2, *CLSTN3* Calsyntenin 3, *SOX10* SRY-box transcription factor 10, *ACSL4* Acyl-CoA synthetase long chain family member 4, *MTF1* Metal regulatory transcription factor 1, *IL-1β* Interleukin 1 beta, *ARE* Antioxidant responsive element, *PHLPP2* PH domain and leucine rich repeat protein phosphatase 2, *MLLT1* Myeloid/lymphoid or mixed lineage leukemia translocated to 1, *PI3K* Phosphoinositide 3-kinases, *Akt* RAC-alpha serine/threonine-protein kinase, *CLCN3* Chloride voltage-gated channel 3, *VEGF-A* Vascular endothelial growth factor A

### Vascular integrity and extracellular matrix remodeling

Maintaining vascular integrity and preserving the extracellular matrix (ECM) are crucial for preventing ICH. Disruptions in the BBB and abnormalities in the ECM contribute to increased vascular permeability and vessel fragility, leading to hemorrhage. The BBB, consisting of ECs, tight junctions, pericytes, and astrocytes, regulates the passage of molecules and cells between the bloodstream and the brain parenchyma [74]. Dysfunction of the BBB is associated with increased permeability, allowing blood components to enter the brain parenchyma, and triggering neuroinflammatory responses. Participation of miRNAs in the regulation of the BBB function occurs largely due to the influence on the cytoskeleton of ECs and on the functional state of cell adhesion proteins [75, 76].

The main cause of ICH is hypertension and associated microangiopathy. Prolonged arterial hypertension contributes to the formation of lipogyalinosis, and subsequently fibrinoid necrosis of the walls of perforating arteries, characterized by the absence of anastomoses with other vessels. Under these conditions, the structure and organization of cell adhesion proteins in the outer membrane of the ECs leads to disruption of BBB permeability (reduced levels of cell adhesion proteins such as claudin 5, occludin and zonula occludens-1 (ZO-1) have been shown) [74, 75]. ZO-1 is an important tight junction protein involved in the formation of the BBB. It is involved in the barrier function, in the regulation of cell transport, cell polarity, cell proliferation and differentiation [77]. An increase or decrease in ZO-1 expression in ECs of the brain microvascular network also significantly affects apoptosis and proliferation of ECs [78]. Hu et al. found that ZO-1 is a potential target for miR-23a-3p. Their data showed that proliferation and apoptosis of hCMEC/D3 cells are regulated by via miR-23a-3p/ZO-1 axis, demonstrating also that aberrant expression of miR -23a-3p promotes the formation of perihematomal edema [59]. In addition, the degradation of cell adhesion proteins by matrix metalloproteinases (MMPs) is also directly regulated by miRNAs [79]. Some miRNAs can protect the integrity of the BBB by reducing immune cell adhesion and the expression of pro-inflammatory cytokines. Activation of microglia in the process of BBB permeability impairment, in turn, exacerbates ICH-induced damage to brain tissue and, thereby, significantly worsens the prognosis and neurological deficit [80, 81]. Thus, maintaining the integrity of the BBB allows minimizing secondary brain damage and the progression of ICH. In this context, miR-27a-3p activation after miR-27a-3p mimic transfection *in vivo* ICH model not only restores BBB function and attenuates neuronal apoptosis, but also reduces microglial activity and leukocyte infiltration in the perihematomal region with improved neurological functions in rats [43].

The ECM provides structural support to blood vessels and plays a role in maintaining their stability. Alterations in ECM components, such as collagen and fibronectin, as well as dysregulated MMPs/tissue inhibitors of metalloproteinases (TIMPs) system, can compromise the integrity of BBB [82]. MMPs and TIMPs are involved in ECM remodeling and degradation, contributing to BBB disruption and increased risk of hemorrhage [82]. Impaired BBB permeability followed by vasogenic edema leads to pronounced expression of matrix metalloproteinases-2 and matrix metalloproteinases-9 (MMP-2 and MMP-9) with damage and disruption of the integrity of the ECM of ECs [83]. At the same time, in IS, degradation of the ECM causes hemorrhagic transformation, which worsens the outcome of stroke [84]. It is known that miR-130a adversely affects BBB permeability, where a decrease in the expression of this miRNA can significantly reduce cerebral edema, reduce BBB permeability, and improve neurological function by increasing the expression of caveolin-1 and reducing the expression of MMP-2 and MMP-9 in an IS model *in vivo* [85]. All this confirms the need to study the molecular mechanisms of the pathogenesis of BBB disorders not only in IS, but also in ICH; where the study of the role of miRNAs in this process could potentially lead to the development of new therapies to prevent secondary brain damage after hemorrhage.

### Inflammation and immune response

Inflammation and immune responses play crucial roles in the pathogenesis of ICH. Hemorrhage in the brain parenchyma triggers an acute inflammatory cascade, characterized by the activation of resident immune cells, such as microglia and astrocytes, and the infiltration of peripheral immune cells. The activated immune cells release pro-inflammatory cytokines, chemokines, and reactive oxygen species (ROS), leading to the recruitment of immune cells and amplification of the inflammatory response [86]. The activation of Toll-like receptors (TLRs), a class of pattern recognition receptors involved in the innate immune response, has been implicated in exacerbating neuroinflammation and neuronal injury in ICH [87]. Targeting TLRs signaling, such as using TLRs antagonists or modulators, has shown promise in preclinical studies by reducing neuroinflammation and improving outcomes [87, 88]. Additionally, the complex interplay between the immune response and the coagulation system contributes to the pathogenesis of ICH [89]. Coagulation factors, such as tissue factor and thrombin, can activate immune cells and modulate the inflammatory response [89]. Targeting specific immune signaling pathways, such as the nuclear factor-kappa B (NF-κB) pathway or the inflammasome, has shown promise in preclinical studies to attenuate neuroinflammation and improve outcomes following ICH [90]. Moreover, recent studies have highlighted the role of neuroinflammation in secondary brain injury after ICH [91].

Microglial activation and the release of pro-inflammatory cytokines, such as interleukin-1β (IL-1β) and tumor necrosis factor-alpha (TNF-α), contribute to neuronal cell death and tissue damage [86]. Modulating the inflammatory response using anti-inflammatory agents, such as minocycline or anti-IL-1β antibodies, has shown promising results in experimental models of ICH [92]. Modulating the inflammatory response using anti-inflammatory miRNAs, such as miR-146a and miR-155, has shown promising results in experimental models of ICH [60, 61]. As mentioned above, the TLRs system is one of the most important factors in the pathogenesis of ICH, demonstrating a high level of expression of Toll-like receptors 2 and Toll-like receptors 4 (TLR-2 and TLR-4) in B cells of patients with ICH [93]. At the same time, miR-146a, known as a negative regulator of the TLR signaling pathway, plays a key role in inflammatory reactions caused by ICH [94]. Since this miR-146a is an essential promoter of macrophage M2 polarization, a high expression level of miR-146 may lead to an improvement in ICH prognosis [60]. Qu et al. in their study demonstrated a significant decrease in miR-146a expression in brain tissues along with a significant increase in the expression of inflammatory molecules (TNF-α and IL-1β) and pro-inflammatory transcripts (tumor necrosis factor receptor associated factor 6 (TRAF6) and NF-κB) and a further deterioration in prognosis [60]. However, intraparenchymal injection of the miR-146a mimic was accompanied by a decrease in the expression of all the above factors and further deterioration of the condition (decrease in neurological deficit and cerebral edema, as evidenced by neurological parameters and water content in the brain). This experiment also revealed the role of miR-146a in the modulation of oxidative stress reactions.

In recent years, many studies have shown that miR-155 plays a regulatory role in inflammatory responses and in the immune response in various human diseases including ICH [95]. In a study in an animal model of ICH, Xu et al. showed increased levels of expression of pro-inflammatory cytokines (interferon-β (IFN-β), interleukin 6 (IL-6) and TNF-α), accompanied by a decrease in the expression level of the suppressor of cytokine signaling 1 (SOCS-1) transcript and an increase in miR-155 expression, indicating a possible role of the miR-155/SOCS-1 signaling pathway in inflammatory process at ICH [61]. In addition, the expression level of miR-155 and pro-inflammatory cytokines significantly decreased after administration of dexamethasone. This suggests that glucocorticoids attenuate the inflammatory process by targeting the miR-155/SOCS 1 signaling pathway in ICH. It has also been demonstrated in other studies that natural and synthetic glucocorticoids are very effective in reducing acute inflammation by downregulating miP-155 expression in a glucocorticoid receptor (GR or GCR) and/or NF-κB dependent mechanism [96, 97]

MiR-155 is known to be NF-κB dependent miRNA [95]. Several central nervous system (CNS) disease studies have shown that during inflammatory responses, the miR-155/NF-kB axis (pro-inflammation) coordinates with the miR-146a/NF-kB axis (anti-inflammation) to regulate the intensity and duration of inflammation [98]. At the same time, miR-146a-deficient cells showed increased expression of miR-155, where the pro-inflammatory phenotype could be attenuated by inhibition of miR-155 expression. The combined action of positive miR-155/NF-kB and negative miR-146a/NF-kB regulatory loops ensures optimal NF-kB activity during inflammatory stimuli and may ultimately lead to resolution of the inflammatory response. Thus, together miR-155 and miR-146a may cross-regulate inflammatory responses and should be properly studied in ICH *in vitro* and *in vivo*.

### Oxidative stress and free radical damage

Oxidative stress, arising from an imbalance between the production of ROS and antioxidant defense mechanisms, is a significant contributor to neuronal injury in ICH. Following a hemorrhagic event, the release of blood components and breakdown products induces the generation of ROS, which can cause oxidative damage to cellular components, including lipids, proteins, and DNA [99]. Lipid peroxidation products, such as malondialdehyde (MDA) and 4-hydroxynonenal (4-HNE), are formed during oxidative stress and can further amplify the inflammatory response and neuronal injury [100]. Antioxidant systems, including superoxide dismutase (SOD), glutathione peroxidase (GPx), and catalase, are essential for neutralizing ROS and maintaining redox homeostasis [101]. Impairment of antioxidant defense mechanisms, coupled with increased ROS production, can lead to oxidative damage and cell death. Several studies have demonstrated the involvement of oxidative stress in the pathogenesis of ICH, highlighting its impact on neuronal cell death and inflammation [98]. In preclinical studies, targeting oxidative stress pathways, such as nicotinamide adenine dinucleotide phosphate (NADPH) oxidase or heme oxygenase-1/2 (HO-1/2), has shown promise in reducing oxidative damage and improving outcomes following ICH [102, 103]. In addition, the study of miRNAs as therapeutic agents in ICH has attracted attention due to their ability to regulate signaling pathways responsible for free radical synthesis and modulation of oxidative stress.

As previously stated, miR-146a plays a role in oxidative stress in ICH by evaluating the expression levels of MDA, SOD, and glutathione peroxidase (GSH-Px) in rat brain tissue [102]. The results showed that, compared with the control group, the expression levels of MDA were increased significantly, the expression levels of SOD and GSH-Px was decreased significantly. In addition, after use miR-146a mimic, these changes were reversed. Xu et al., investigated the protective effects of miR-27b inhibition on ICH *in vivo* [104]. In particular, the authors have shown that intracerebroventricular (ICV) injection of anti-miR-27b attenuated the ICH-induced oxidative damage and neuroinflammation in the experimental ICH rat model. To understand whether miR-27b inhibition increases the antioxidant and anti-inflammatory capacity of nerve cells, the authors assessed ROS production, MDA and TNF-α expression in PC12 cells. It was shown that increased ROS production was reduced by anti-miR-27 transfection and the effect was blocked by nuclear factor erythroid 2–related factor 2 (Nrf2) knockdown. In addition, elevated levels of MDA and TNF-α expression were reduced by anti-miR-27b transfection. As a result, the conclusion is that miR-27b inhibition diminished ICH-induced oxidative and inflammatory injury, raising the prospect of using miR-27b inhibition as a therapeutic strategy for ICH. The intricate interplay between oxidative stress, antioxidants, redox signaling, and their regulation by miRNAs in ICH necessitates further investigation for the development of targeted antioxidant therapies.

## MiRNAs as possible therapeutic agents

Currently, the importance of miRNAs as potential targets for targeted therapy in stroke is being actively discussed. Several approaches have been proposed to control miRNA expression in animal models of IS and ICH chemically modified stable nuclease-resistant oligonucleotides have been developed [46–48]. In several studies in animal models of IS, targeting of LNA (Locked nucleic acid) or 2'-O-methylated antisense oligonucleotides on individual miRNAs was effective in reducing their expression, inhibiting the pro-inflammatory response, and reducing acute brain injury [104, 105].

The potential therapeutic effect on miRNAs (including blocking miRNAs expression or introducing potentially useful miRNAs) may depend on several important factors, including the efficiency of delivery systems to target organs/tissues. It has been suggested that the efficiency of miRNA delivery into the cell is determined by interaction with lipoprotein particles, lipoprotein receptors, and transmembrane proteins. The small size of these molecules, which are filtered and excreted by the kidneys, also reduces the efficiency of delivering these therapeutic molecules from the bloodstream to target tissues. To solve this problem, pegylated liposomal forms and/or biodegradable polymers are used [106]. In addition, to avoid filtration by the kidneys, these molecules are enclosed in liposomes, which, due to their large size (> 50 Da), do not pass through the glomerular barrier [106]. Despite the progress achieved in translational studies, further refinement of the specificity of the effect of therapy aimed at modulating the effects of miRNAs in specific tissues/organs on other systems and organs, as well as clinical studies of their safety and toxicity, is necessary.

### Methods for preclinical evaluation of side effects of miRNA-based therapy

Evaluation of side effects of test compounds, including miRNAs, on cell lines and experimental animals is a mandatory step in the development of new drugs. The results obtained during studies on cells and animals are used to assess the feasibility of promoting miRNA candidates for further development of miRNA-based therapy, the design of the first stage of clinical trials and selection of the initial dose. Despite the fact that studies on experimental animals are the “gold standard” for preclinical evaluation of the safety of miRNA-based therapy, this approach has significant drawbacks: 1) Due to the species specificity of miRNAs and the fact that each miRNA can control many (up to several hundred) genes, at the same time, one mRNA can carry many binding sites for one or different miRNAs; 2) the number of animals used to assess the safety of the compounds and the duration of the studies do not allow the detection of rare and long-term adverse effects; 3) due to the cost of experiments, time costs and the requirements of ethical committees, it is necessary to reduce the number of animals used at the stage of preclinical studies, which contradicts the safety requirements of drugs under development [3, 107].

Cell cultures are widely used in drug development to evaluate the metabolism, genotoxicity, and mutagenicity of test compounds. The same approach can also be used to assess the therapeutic and adverse effects of miRNA-based therapy. Co-cultures of cells containing multiple cell types, such as peripheral blood mononuclear cells (PBMCs) and ECs, are also widely used [108]. Co-cultures of cells model the interaction of the respective cell types in human tissues and organs (e.g., the CNS and its structures), which allows for a more accurate assessment of the effects of the compound *in vivo*. However, there are disadvantages. First, the cell lines used may differ in phenotype from the corresponding cells *in vivo*. For example, hepatocytes lack the glucagon signaling pathway, and ECs lack expression of receptor ligands associated with lymphocyte homing. Second, not all cell types are commonly available as self-replicating cell lines. Thirdly, the method using joint cell cultures, and even more so cultures of individual cell types, does not allow us to study more complex effects *in vivo* associated with the interaction of cells, tissues, and organs through neurohumoral mechanisms, for example, disruption of the central mechanisms of blood pressure regulation [109, 110]. This means that work with cell cultures does not allow one to reveal many adverse effects of miRNA-based therapy, in particular, the mechanisms of side effects.

### Problems of and ways to solve them for application miRNA-based therapy

The use of miRNAs in therapeutic practice has significant limitations: sensitivity to blood serum nucleases, the possibility of nonspecific binding, actions by the small interfering RNA (siRNA) mechanism, which leads to suppression of the expression of genes other than the target, the mRNA of which is partially complementary to the “seed” region, and activation of the innate immune response [110]. To achieve a therapeutic effect upon systemic delivery, miRNAs molecules must be in an active form during circulation in the bloodstream, and avoid filtration by the kidneys, absorption by phagocytes, formation of aggregates with blood serum proteins, and degradation by nucleases. In addition, to penetrate the brain parenchyma (e.g., to reduce neuronal apoptosis and eliminate the inflammatory process), miRNA guide chains must pass through the BBB [111]. This barrier traps molecules larger than 5 nm. However, the vessels of the liver and spleen pass molecules up to 200 nm in diameter, and the vessels of tumors - substances with a molecular weight of more than 40 kDa [112]. This phenomenon is known as enhanced permeation and retention (EPR) effect. However, as in the case of ICH, there is a violation of the barrier function of the BBB, which allows some molecules, including miRNA agents, to enter the CNS. Or, in the case when circulating miRNAs are considered as non-invasive biomarkers, miRNAs can penetrate the blood or cerebrospinal fluid (CSF) from the CNS. For instance, Sørensen et al., demonstrated that miRNAs can freely cross the BBB and circulate freely in the bloodstream and CSF in patients with IS and multiple sclerosis [113]. In this study, half of the stroke patients had a high serum albumin (Qalb) value. Three patients with multiple sclerosis also had a clearly elevated Qalb. This indicates that a significant proportion of these patients had changes in the function of the BBB. Changes in the CSF/Qalb ratio are currently recognized as one of the most reliable indicators of impaired BBB permeability.

**Fig. 2:**
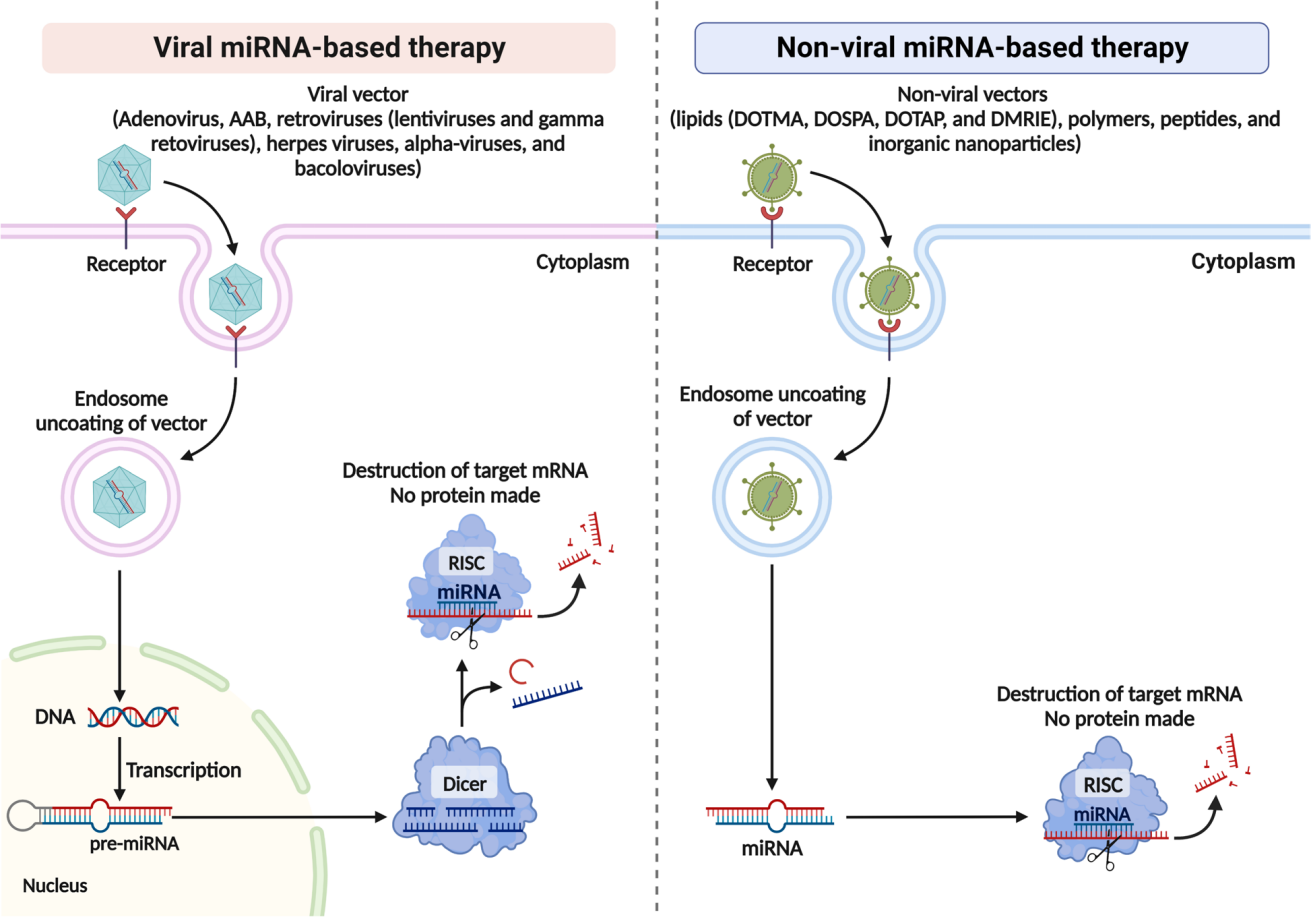
Types of vector delivery systems for microRNAs (miRNAs) agents for potential therapy in non-traumatic intracerebral hemorrhage (ICH) models *in vitro* and *in vivo.* There are viral and non-viral miRNA delivery systems. The achieved level of last research makes it possible to create vector systems that target different types of cells of the central nervous system (CNS) (e.g. adenoviral vectors can infect neurons, astrocytes, oligodendroglia, ependymocytes, choroidal epithelium, and microglia *in vivo*) and direct miRNAs directly to the target. Note: pre-miRNA, precursor microRNA; RISC, RNA-induced silencing complex; mRNA, messenger RNA; DOTAP, 1,2-dioleoyl-3-trimethylammonium-propane; DOTMA, 1,2-di-O-octadecenyl-3-trimethylammonium propane; DOSPA, polycationic lipid 2,3-dioleyloxy-N-[2(sperminecarboxamido)ethyl]-N,N-dimethyl-1-propanaminium trifluoroacetate; DMRIE, N-( 1 ,2-dimyristyloxyprop-3-yl)-N,N-dimethyl-N- hydroxyethyl ammonium bromide

As already mentioned, BBB permeability is disturbed during ICH and this may allow some delivery of miRNA agents through the blood circulation, but there is also a need for delivery directly to CNS cells. After miRNA molecules leave the bloodstream, they must pass through the ECM cells (e.g., neurons), the network of structural proteins and polysaccharides surrounding the target cells [114]. ECM can significantly hinder the uptake of miRNAs by cells, thereby increasing their likelihood of phagocytosis and cleavage. The plasma membrane is the main barrier for miRNA penetration into the cell. The hydrophilic nature, high molecular weight, as well as the total negative charge of miRNA molecules determine the low efficiency of their absorption [115]. There are several ways to solve this problem. For example, the combination of miRNA molecules with cationic lipids and polymers leads to the neutralization of the negative charge of miRNA and the formation of positively charged complexes [116]. It has been shown that non-viral carriers enter cells by endocytosis. There is clathrin-mediated endocytosis, caveolar-mediated endocytosis, macropinocytosis, as well as clathrin- and caveol-independent endocytosis [117]. Unlike viruses, non-viral vectors (synthetic vectors) are characterized by low transfection efficiency (Fig. 2).

One of the ways to increase the uptake of carriers by cells is the attachment of specific ligands that promote receptor-mediated endocytosis of transport molecules. Such ligands typically target receptors that mediate nutrient absorption: transferrin, folic acid, and low-density lipoprotein receptors [118]. After entering the cell, miRNA molecules are found in early endosomes. Owing to the work of vacuolar H+-ATP-ase, the environment of early endosomes is acidified (pH decreases to 5–6), because of which they are converted into late endosomes. Then, late endosomes fuse with lysosomes, which have even lower pH values (pH ~ 4.5) and contain miRNA-cleaving nucleases [119]. To avoid degradation in lysosomes, miRNA molecules (in unbound form or in complex with the carrier) must exit the endosome into the cytosol. Exit from the endosome is the main limiting step in the process of RNA interference (RNAi) [118, 119].

Efficient miRNA delivery using various cationic polymers is due to the large buffer capacity of these compounds (due to non-protonated secondary and tertiary amines) in the pH range from 5 to 7. It is assumed that such polymers prevent acidification of the endosome environment by acting as proton sponges. In this case, there is an increase in the influx of protons due to the activation of the vacuolar H+-ATP-ase, accompanied by the accumulation of Cl- anions, as well as an increase in osmotic pressure. This leads to osmotic swelling and disintegration of the endosome. The release of vectors based on cationic lipids from endosomes is mediated mainly by electrostatic interactions of these molecules with negatively charged phospholipids of endosome membranes, as well as by the ability of lipid structures to pass from the lamellar phase (bilayer) to the hexagonal one. The formation of cation-anion pairs destabilizes the lipid bilayers, resulting in the release of nucleic acids from the complex [119–121].

## The need for biomarkers for ICH

Modern stroke diagnosis is based on clinical examination data and neuroimaging techniques. Liquid biopsy looks for single biomarkers or multiple sets of them that can be used to diagnose acute stroke, to differentiate types of strokes (e.g., non-traumatic hemorrhage from ICH due to ruptured intracranial aneurysms (IAs)), or even to predict initial or recurrent stroke, may be very valuable. Modern diagnosis of ICH is difficult, diagnosis appears to be delayed due to the lack of a suitable mechanism for a rapid (ideally at the patient's bedside), accurate, and analytically sensitive diagnostic method based on the determination of biomarkers. There is a clear need for further research in this area. Potential biomarkers require rapid clinical confirmation of their use for early diagnosis, real-time diagnosis, and prognosis ICH, which should improve the outcome of the disease and the quality of life of patients.

Considering the prevalence and variety of clinical manifestations of stroke, a promising direction is the development of prognostic criteria for assessing the severity of this pathology, the possible restoration of lost functions, and determining the risk of recurrent hemorrhage. Predicting the course and outcome of stroke is important for clinicians, patients, and researchers. Despite the large number of studies devoted to the search for prognostic biomarkers for stroke, this problem remains relevant [122]. In recent years, the method of determining the concentration of biomarkers in whole blood, plasma/serum, and CSF, which can enable practitioners to diagnose and predict the course of stroke development, has become increasingly popular [122, 123]. The use of a panel of certain biomarkers, reflecting the complex interactions of biochemical and pathophysiological processes occurring in this pathology, can provide more accurate information about the severity of hemorrhage and brain damage, determine the patient's reparative capabilities, which will make it possible to predict the outcome of ICH in the early stages of the course of the disease and implement the most effective treatment tactics.

Difficulties in the discovery of biomarkers are associated with the slow penetration of glial and neuronal proteins through the BBB after a stroke or traumatic brain injury (TBI) [124]. In addition, markers of cerebral ischemia or cerebral vessel injury may lack diagnostic specificity and increase in some cases of mimicking IS/ICH [125]. Ideal biomarkers should have characteristics that include diagnostic specificity and sensitivity to stroke, the ability to differentiate between ICH and IS, the ability to release early and sustainably immediately after a stroke, predictive ability, potential for risk assessment and guiding therapy, and the ability to be qualitatively and rapidly measured using cost-effectiveness technologies.

## Traditional biomarkers of ICH and circulating miRNAs

**Fig. 3:**
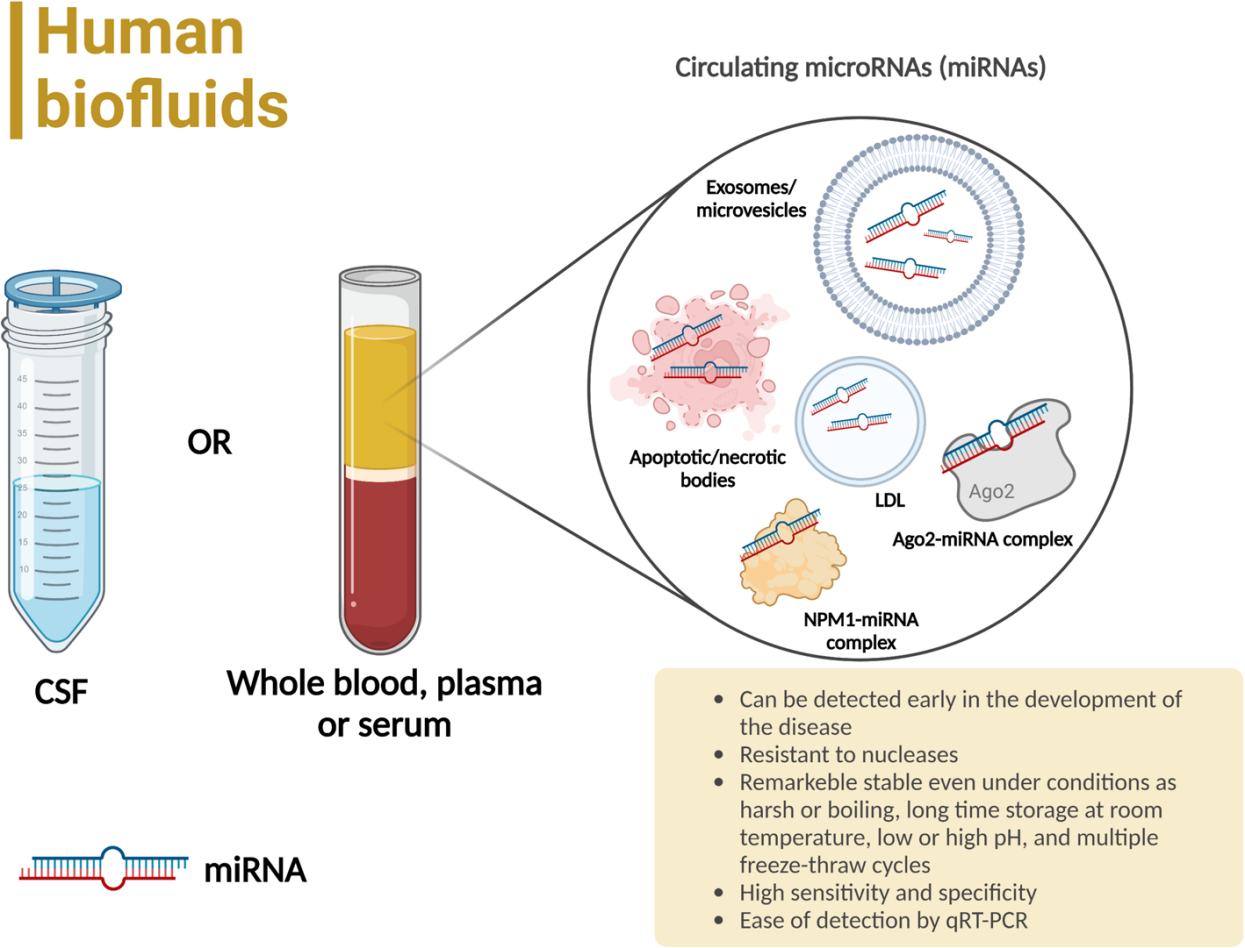
Possibilities of using circulating microRNAs (miRNAs) as non-invasive biomarkers. The most convenient biological fluids for detecting changes in the expression of circulating miRNAs in non-traumatic intracerebral hemorrhage (ICH) are blood (whole blood or plasma/serum) and cerebrospinal fluids (CSF). Detection in CSF is especially promising in patients with complicated ICH such as ICH with penetration into the brain lateral ventricles. Note: LDL, Low-density lipoproteins; Ago2, Argonaute 2; NPM1, Nucleophosmin 1; CSF, Cerebrospinal fluid

**Fig. 4:**
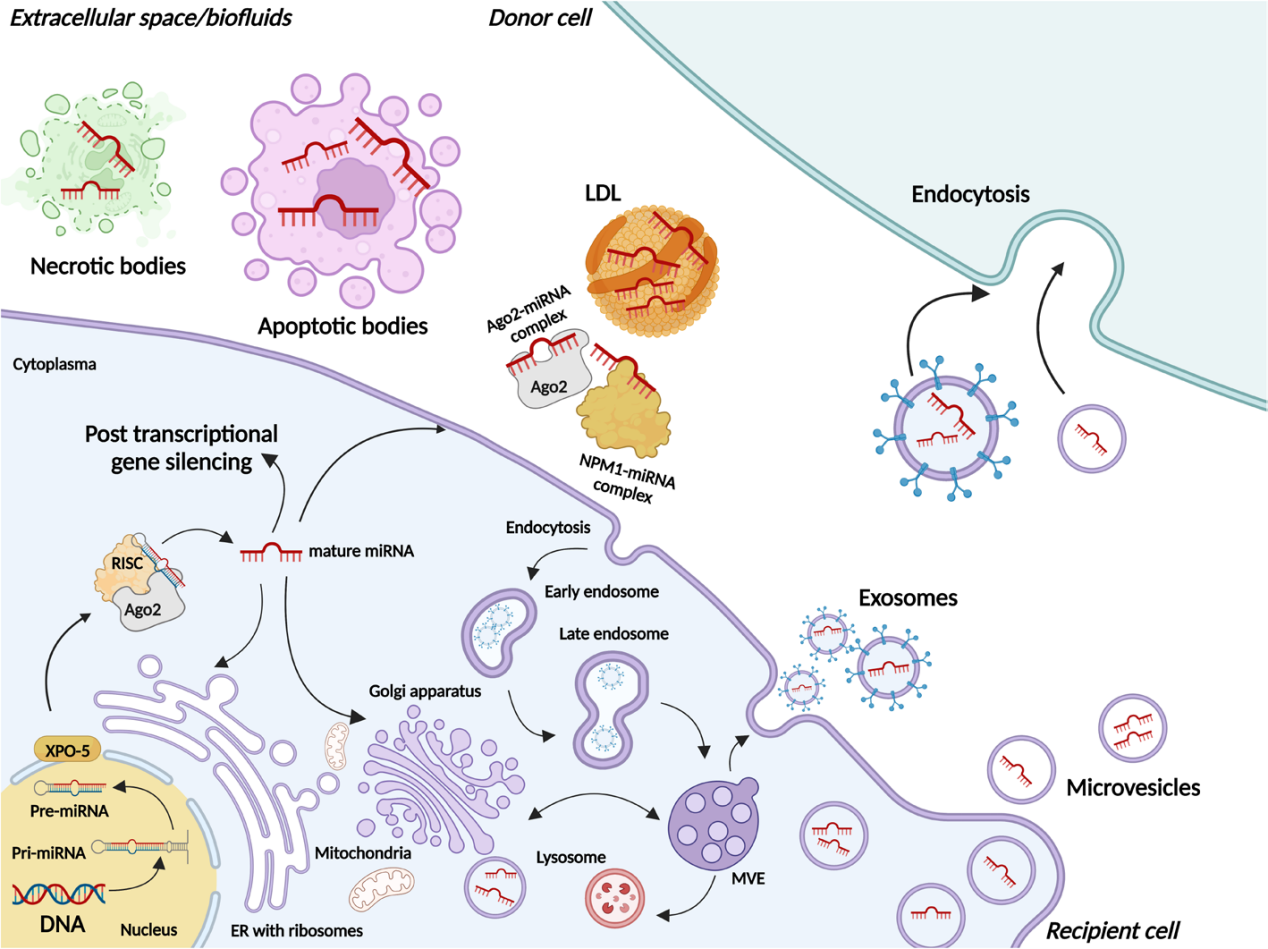
Types and pathways of microRNA (miRNAs) secretion into the extracellular space/biofluids. MiRNAs that can be secreted from cells are called circulating or extracellular or cell-free miRNAs. To date, two secretion pathways are known: 1) passive secretion from damaged cells due to apoptosis or necrosis and 2) active secretion with the help of extracellular vesicles (EVs), including exosomes and microvesicles (MVs), as part of low-density lipoproteins (LDL) or using an RNA-binding protein-dependent pathway (complex of miRNA-Argonaute 2 (Ago2) or miRNA- nucleophosmin (NPM1)). Note: pri-miRNA, primary microRNA; pre-miRNA, precursor microRNA; XPO5, Exportin 5; RISC, RNA-induced silencing complex; ER, Endoplasmic reticulum; MVE, Multivesicular endosomes

Biomarker analysis is carried out in various biological fluids. As has already been demonstrated in many works, biomarker levels are assessed using indicators of accuracy, sensitivity, and specificity [126]. It is important to know that many biomarkers should not be expected to have high sensitivity and specificity at the same time. Often an indicator with high sensitivity can provide a confirmatory analysis of the presence of a particular disease. To date, the most studied and used in clinical practice are protein biomarkers of stroke [127]. A convenient way to categorize stroke biomarkers is to divide them by origin into groups: markers of brain tissue damage, inflammatory, hemostatic, lipid metabolism, and other markers [127]. However, there are many disadvantages such as instability or disease specificity. Circulating miRNAs can be used as potential biomarkers for predicting, detecting, identifying, and monitoring ICH, since their expression profile specifically changes when pathology of cerebral vessels occurs. Estimation of the content of circulating miRNAs is currently one of the most promising areas of molecular diagnostics. This is explained by miRNA resistance to degradation, specificity, ease of detection, etc. (Figs. 3 and 4) [3, 128, 129]. In addition, as is already known, existing proteins as ICH biomarkers (for example, vascular cell adhesion molecule 1 (VCAM-1), brain natriuretic peptide (BNP) and MMP-9) involved in microglial activation, endothelial dysfunction, cell metabolism disorders, etc., the expression of which should be very clearly regulated in the cell by miRNA. That is, the relationship between miRNA and 3'-UTR mRNA of target genes encoding these same proteins [3, 130]. Table 4 lists several biomarkers that can be used in the context of ICH; in addition, these data also demonstrate the regulatory role of certain miRNAs for one or another biomarker of ICH [131–138].

**Table 4:** The results of studies demonstrating the role of several microRNAs (miRNAs) in the regulation of the expression of some protein biomarkers in non-traumatic intracerebral hemorrhage (ICH) are presented

**Biomarker**	**Type of biomarker**	**Function of biomarker**	**miRNA**	**Reference**
MMP-9	Inflammatory and brain damage	MMP-9, also known as gelatinase B, is produced by astrocytes in response to inflammation and is involved in primary ICH	MiR-130a, miR-126-3p and miR-18a	[41, 46, 47, 131]
GFAP	Brain damage	Identified as a promising candidate for point-of-care analysis indicative of ICH in the early stages of disease. GFAP is rapidly released from damaged glial cells because of an expanding hematoma	MiR-126	[39, 132]
D-dimer	Hemostasis	D‐dimer as a blood biomarker assessing late clinical severity of ICH may add early prognostic information and be a suitable target	MiR-145	[133, 134]
TNF-α, IL-1β and IL-6	Inflammation	Cytokines are diagnostic markers for the prognosis and outcome of ICH patients	MiR-132, miR‐21‐5p, miR-331-3p, miR-378a-5p, miR-146a, and miR‑155	[39, 42, 45, 52, 53, 60, 61, 135]
NSE	Brain damage	NSE is released into the systemic bloodstream after brain damage, including ischemic stroke/ICH, traumatic or hypoxic damage, and NSE is relatively specific to neurons	MiR-20a-3p and miR-145	[136, 137]
VCAM-1	Inflammatory and vascular damage	VCAM-1 is a protein that is canonically involved in the adhesion and transmigration of leukocytes to the interstitium during inflammation including in cerebral vascular and brain tissue	MiR-126-3p and miR-126-5p	[8, 44]
MCP-1	Inflammatory and vascular damage	MCP-1 is a biomarker reflecting the formation of inflammatory infiltrates in the walls of cerebral vessels. MCP-l provides activation of cellular immunity and migration of phagocytes to the focus of inflammation. Determination of the MCP-1 level may reflect the severity of endothelial dysfunction	MiR-221	[138]

*Abbreviations*: *MMP-9* Matrix metalloproteinase 9, *GFAP* Glial fibrillary acidic protein, *IL-6* Interleukin 6, *TNF-a* Tumor necrosis factor alpha, *IL-1β* Interleukin 1β, TNF-a, Tumor necrosis factor alpha, *NSE* Neuron-specific enolase, *VCAM-1* Vascular cell adhesion molecule-1, *MCP-1* Chemoattractant protein -1

## Circulating miRNAs as biomarkers for ICH

**Fig. 5:**
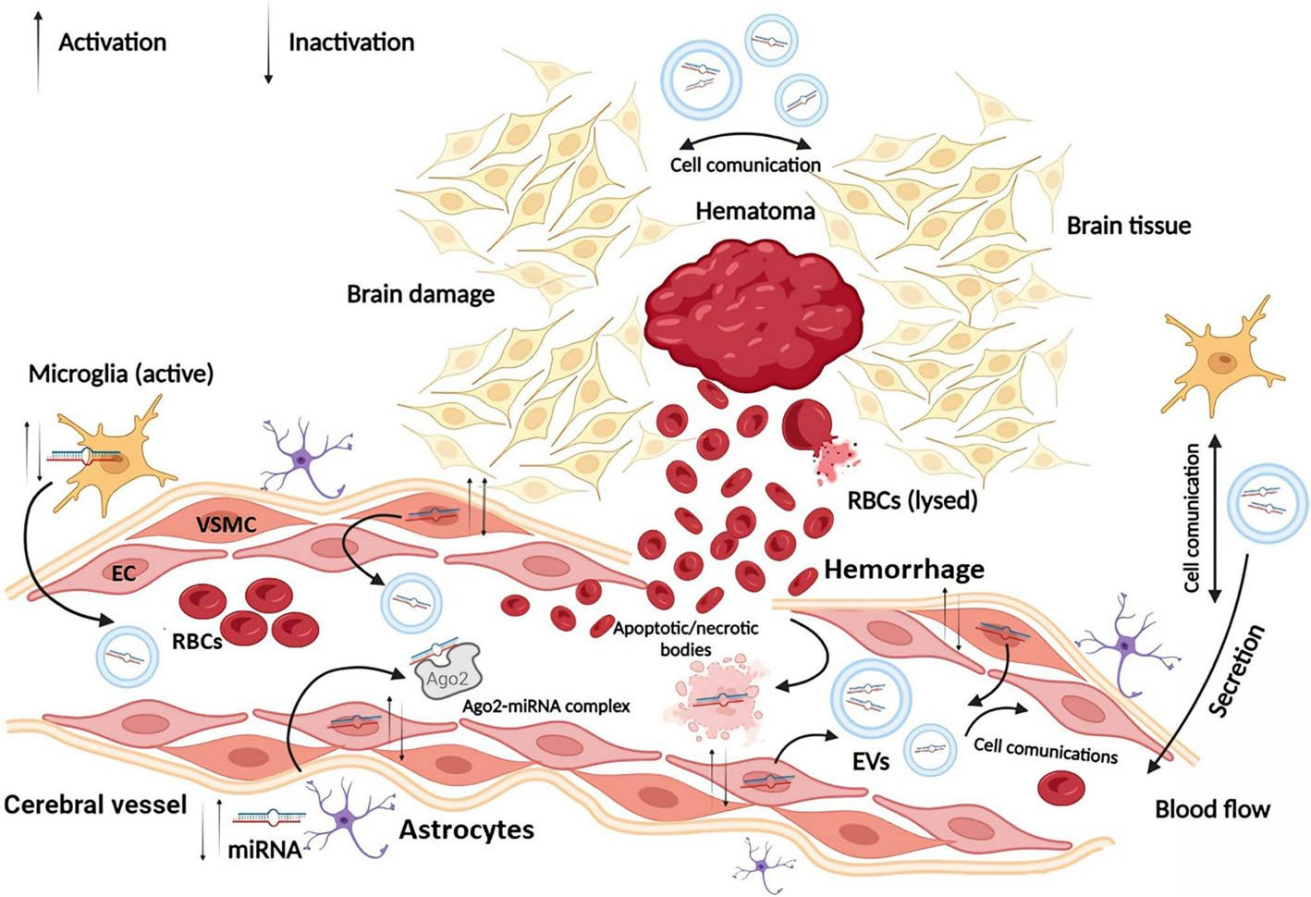
Schematic illustration of microRNAs (miRNAs) secretion from cells of the central nervous system (CNS) into the extracellular space/biological fluid (blood or cerebrospinal fluid (CSF)) after non-traumatic intracerebral hemorrhage (ICH). Various forms of circulating miRNAs are presented: 1) in the composition of apoptotic/necrotic bodies after the death of intima cells (endothelial cells (ECs) and vascular smooth muscle cells (VSMCs)) of a cerebral vessel because of prolonged damage to the vessel (before hemorrhage) and after its rupture (after hemorrhage); after the death of neuronal cells due to hematoma compression (secondary ischemic damage) or due to lysis of erythrocytes (miRNAs of erythrocyte origin. 2) In the composition of extracellular vesicles (EVs) (exosomes or microvesicles (MVs)) responsible for cellular communication between vascular cells or microglial cells - the progression of the inflammatory response and endothelial dysfunction. 3) as part of protein complexes (see Figs. 3 and 4). Note: Ago2, Argonaute 2; RBCs, Red blood cells (erythrocytes)

The main cause of ICH remains the weakening of blood vessels associated with chronic hypertension and atherosclerotic damage; despite some controversy, there is no significant prevalence of ICH, despite the frequent use of anticoagulants. The onset of ICH symptoms can also be gradual or rapid, and the clinical outcome is highly dependent on the size and extent of the hematoma [139]. During the first few hours after ICH, varying degrees of edema occur, followed by retraction of the blood clot and release of osmotically active proteins into the surrounding tissues. After 2-3 days, activation of the coagulation cascade occurs in combination with thrombin synthesis and a systemic inflammatory response. Finally, hemoglobin toxicity to neurons and erythrocyte lysis occurs, which occurs within days of the initial attack of ICH [3, 140, 141]. The search for biomarkers in the face of circulating miRNAs of ICH is especially important due to the delayed destruction of the BBB, which remains intact for large molecules for several hours after a hemorrhage (Fig. 5). Analyzing the ways of spreading the pathological process, it is necessary first to consider the mechanisms of intercellular communication. Traditionally, three main types of communication were considered between the cells of the CNS: 1) signal transmission through chemical synapses using neurotransmitters; 2) cell interaction through gap junction channels; and 3) paracrine regulation, which involves several different mechanisms [142]. In recent years, an increasing role has been assigned to the communication of cells of the CNS using the fourth type of interactions – extracellular vesicles (EVs), represented by exosomes and microvesicles (MVs) (Fig. 5) [143]. EVs occupy an important place in intercellular communication in the CNS both in normal conditions and in ICH, primarily performing several functions associated with the transport of miRNAs (exosomal miRNAs) and the regulatory role of miRNAs in signaling pathways in donor cells [50, 144]. In addition, the encapsulation of miRNAs in EVs protects them from degradation and dissolution in the extracellular space, which allows them to be delivered over long distances in the human body. They can freely move in the intercellular environment and are found in any biological fluids [19]. In the CNS, EVs are secreted into the extracellular environment by neurons, microglia, astrocytes, and oligodendrocytes, as well as ECs and VSMCs of cerebral vessels. Neuronal EVs are located mainly in the somatodendritic regions, where the probability of their detection is 50 times greater than in axons [145, 146]. Considering all these factors, circulating miRNAs in EVs are most often considered as non-invasive biomarkers, in particular, for possible prevention, diagnosis, prognosis, and monitoring of therapy of ICH patients. In Table 5, we summarized all possible studies conducted on the study of circulating miRNAs as potential biomarkers in ICH [41, 43–45, 49, 58, 59, 147–160]

**Table 5:** Clinical and preclinical studies on the study of circulating miRNAs as non-invasive biomarkers in non-traumatic intracerebral hemorrhage (ICH)

**miRNAs**	**Regulation**	**Number of patients, n**	**Animals, n**	**Biofluid**	**AUC/ LASSO**	**Sensitivity, %**	**Specificity, %**	**Important find**	**References**
MiR-130a	Up	50	-	Serum	-	-	-	Associated with severe perihematomal edema and predict adverse outcome	[41]
MiR-27a-3p	Down	26	6	Serum	-	-	-	Predictive of ICH and prognosis of neurological deficits (NIHSS scores)	[43]
MiR-126	Down	38	48	Serum	-	-	-	Predictive of ICH and prognosis of neurological deficits (NIHSS scores)	[44]
MiR‐21‐5p	Up	48	-	Serum	-	-	-	Correlates with the neurological deficit severity (NIHSS scores) and clinical outcomes (mRS scores at 90 days) of elderly patients	[45]
MiR‑29a	Down	-	240	CSF	-	-	-	Post-ICH prognosis	[49]
MiR-124-3p	Down	48	-	Serum	-	-	-	Correlates with the clinical outcomes (mRS scores at 90 days) of elderly patients	[58]
MiR-23a-3p and miR-130a	Up	30	-	Serum	-	-	-	miR-23a-3p is positively correlated with PHE volume	[59]
MiR-26a and miR-146a	Down								
MiR-126, miR-146a, miR-let-7a, and miR-26a	Down	33	-	Serum	-	-	-	miR-126 expression levels positively correlate with PHE, indicating its potential usefulness as a biomarker for PHE management	[147]
MiR-145	Up	106	-	Plasma	0.766	-	-	miR-181b and miR-145 had considerable accuracy in discriminating the plasma of patients with ICH from that of the control group	[148]
MiR-223	Up				0.78				
MiR-155	Up								
MiR-181b	Down								
MiR-27a, miR-365, miR-150, and miR-34c-3p	Up	11	-	Plasma	-	-	-	Bioinformatics analysis of the present data suggested that these circulating miRNAs species were specifically enriched in biological processes of inflammatory disease and inflammation response.	[149]
MiR‐1249, miR‐574‐5p, miR‐1290, miR‐522, miR‐130a, miR‐1202, let‐7f‐2, miR‐586, miR‐122, and miR‐29c	Up	79	-	Plasma	1.0	100	90.0	The results indicate that these miRNAs can be used as biomarkers in the prediction of hematoma growth	[150]
MiR-124	Up	20	18	Plasma	-	-	-	miR-124 is brain tissue specific miRNA. ​Plasma miR-124 pattern and concentrations in the detection, diagnosis, and prognosis of human ICH patients	[151]
MiR-124-3p, miR-137-5p, miR-138-5p, miR-219a-2-3p, miR-135a-5p, miR-541-5p, and miR-770-3p	Up	-	3	Serum	-	-	-	The panel of miRNAs may have roles in the pathophysiology of ICH in the elderly. Among those, miR-124-3p, miR-138-5p, and miR-137-3p may have the greatest potential, as they are human-brain-specific miRNAs and are also implicated in neuronal apoptosis and neuroinflammation	[152]
MiR-1183 and let-7d-3p	Up	23	-	Whole blood	-	-	-	The panel of miRNAs may identify as predictive biomarkers	[153]
MiR-4707, let-7i, miR-146a-5p, miR-17-3p, miR-210-5p and miR-221	Down								
MiR-4325 and miR-4317	Down	6	-	Whole blood	-	-	-	The panel of miRNAs was identified as a potential predictor of post-ICH late seizure.	[154]
MiR-181a-5p and miR-1180-3p	Up								
MiR-211-3p	Up	17	-	Serum	-	-	-	Suggested that miR-211-3p is an immune-related biomarker and may prognosis in the secondary injury following ICH	[155]
20 miRNAs	Up and down	19	-	Plasma	0.824	-	81.0	miRNAs have predictive value, and could be capable of distinguishing between major stroke (ICH, SAH and IS) subtypes with refinement and validation	[156]
MiR-150-5p and miR-v-5p	Up	-	55	Plasma	-	-	-	The expression levels of miRNAs in blood have been found to be reproducible and indicative of the disease state.	[157]
MiR-223, miR-155, miR-181b, and miR-126	Up	47	-	CSF	-	-	-	As predictive biomarkers of possible ICH complications such as IVH. In additional, effectively monitor inflammatory conditions as potential biomarkers in preterm IVH	[158]
MiR-21-5p	Down	31	-	Serum	-	-	-	Important role to play in the prognosis of patients with ICH. Downregulation of miR-21 expression in the peripheral blood may correlate with pathological changes (neuron apoptosis, inflammation, and the occurrence of cerebral edema)	[159]
MiR-124-3p	Up	93	-	Plasma	0.70	68.4	71.2	Plasma miR-124-3p levels were markedly higher in patients with ICH patients compared to IS patients. MiR-124-3p is useful in monitoring disease progression in ICH, such as haematoma expansion	[160]

*Abbreviations*: *CSF* Cerebral spinal fluid, *AUC* Area under ROC curve, AUC and LASSO analyses is considered diagnostically significant for the biomarker, - Not mentioned in the article, *NIHSS* National Institutes of Health Stroke Scale, *mRS* Modified Rankin Score, *PHE* Perihematomal edema, *SAH* Subarachnoid hemorrhage, *IS* Ischemic stroke, *IVH* Intraventricular hemorrhage, *CT* Computed tomography

### Circulating miRNAs as biomarkers of early damage to the vascular wall

If we return to the issue of the pathogenesis of ICH, then a special topical problem was the allocation of a separate area of research on early damage to the vascular wall of cerebral vessels. The literature is actively discussing the need to search for diagnostic tools or indicators of early damage to the cerebral arteries to use them as biomarkers that can predict its rupture. Chronic hypertension and atherosclerosis together correspond to damage to the endothelium and ECM and subsequent remodeling processes in the form of thickening of the intima-media complex (IMC) and fibrosis of the cerebral artery wall [161]. The unique location of the endothelium makes it sensitive to changes occurring both in the systemic circulation and in tissues. Following intima injury, ECs, immune system cells, and platelets release several regulatory factors such as growth factors and cytokines (see below) that change the contractile to synthetic phenotype and increase VSMCs proliferation and migration, leading to the formation of a new hyperplastic intima (neointima) [3, 139–141].

**Fig. 6:**
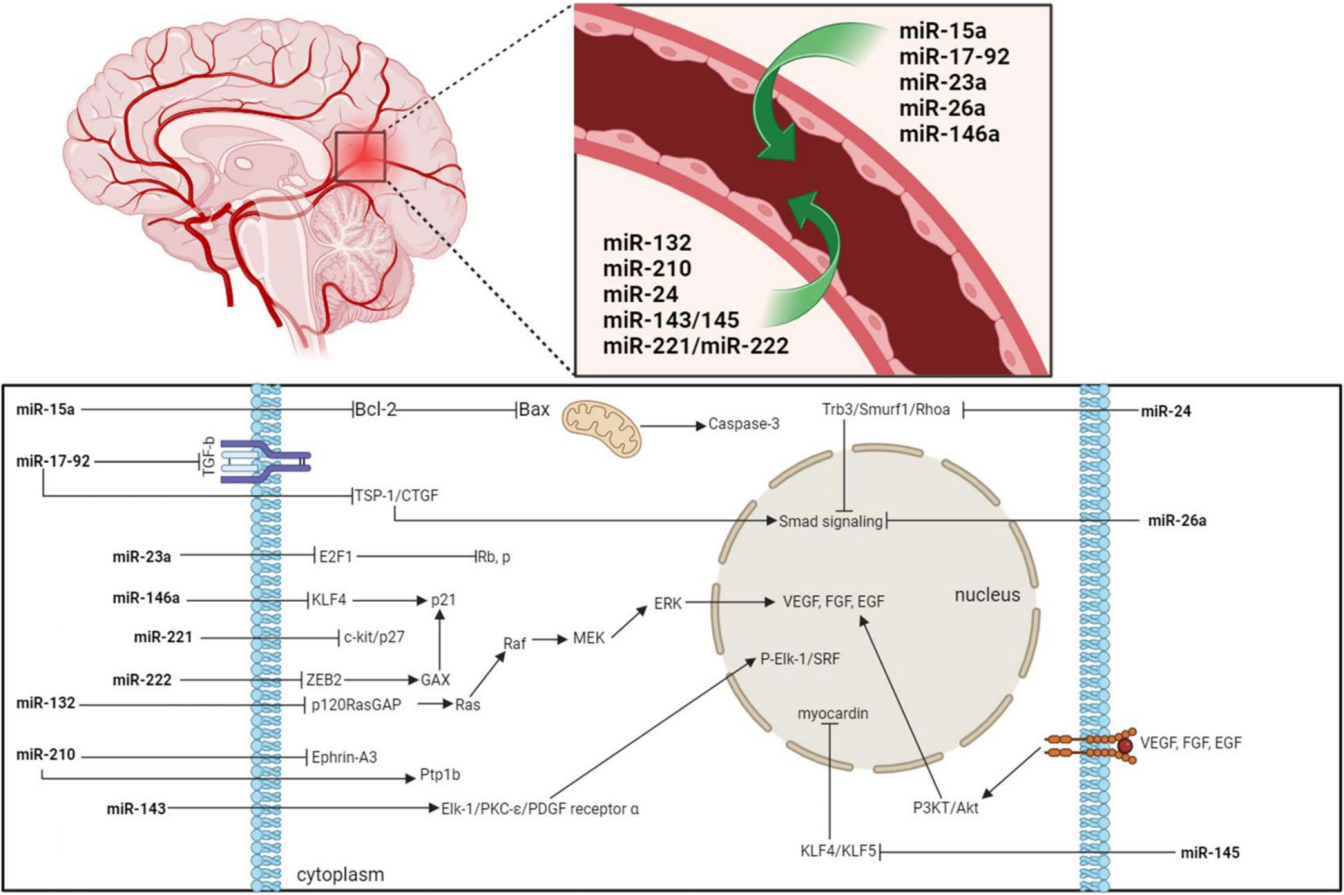
Schematic illustration of the regulatory role of microRNAs (miRNAs) in vascular wall cells (endothelial cells (ECs) and vascular smooth muscle cells (VSMCs)) and their secretion into the bloodstream. Vascularly enriched miRNAs regulate cellular responses to various factors both in normal and pathological conditions by acting on various receptors and signaling pathways: miR-15a - decreases blood-brain barrier (BBB) disruption and protection of cerebral ECs apoptosis; miR-17-92, miR-210 and miR-132 - angiogenesis; miR-23a - inhibition of ECs growth; miR-26a - increase VSMCs proliferation and control VSMC phenotype shifting; miR -143/145 and miR-146a - activation of VSMCs proliferation and neointimal hyperplasia; miR-24 - inhibition of erythropoiesis and control VSMC phenotype shifting; miR-221/miR-222 - pro-proliferative effect on ECs and angiogenesis. In addition, it is known from recent sources that these vascular-enriched miRNAs can occur in the bloodstream and may be biomarkers of early damage to the vascular wall. Note: Bax, Bcl-2-associated X protein; Bcl-2, B-cell lymphoma 2; CTGF, connective tissue growth factor; EGF, epidermal growth factor; Elk-1, ETS domain-containing protein 1; ERK, extracellular signal-regulated kinase; FGF, fibroblast growth factor; GAX, homeobox protein; KLF4, krüppel-like factor 4; KLF5; krüppel-like factor 5; MEK, mitogen-activated protein kinase kinase; PI3K, Phosphatidyl-inositol 3-kinase; p120RasGAP, p21, cyclin-dependent kinase inhibitor 1; Rb, Retinoblastoma; p, Hpophosphorylated; Raf, rapidly growing fibrosarcoma; Ras, rat sarcoma; RhoA, Ras homolog gene family member A; Smurf1, Smad ubiquitin-regulatory factor 1; SRF, serum response factor; TGF-b, transforming growth factor beta; Trb-3, Tribbles-like protein-3; TSR1, thrombospondin-1; VEGF, vascular endothelial growth factor; ZEB2, zinc finger Ebox- binding homeobox 2.

The occurrence and development of endothelial disfunction is influenced by oxidative stress, the synthesis of vasoconstrictors and cytokines that suppress the production of nitric oxide (NO), the main vasodilator [99, 100]. The resistance of the vascular wall to the action of damaging factors depends on the stability of the cell membrane of ECs and the integrity of intercellular contacts. Thus, cell activation and apoptosis lead to morphological changes in ECs, changes in the rheological properties of blood, cause desquamation of ECs, and stimulate platelet adhesion and aggregation [162]. Due to the violation of intercellular contacts, the permeability of the endothelium for lipoproteins and monocytes increases and, thus, the development of atherosclerotic damage to the vascular endothelium begins [1, 139]. In this regard, fibrinogen, C-reactive protein (CRP), homocysteine was classified as newly specified risk factors/biomarkers in ICH, and IL-6, TNF-α, IL-1β, lipoprotein-associated phospholipase A2 (Lp-PLA2), cyclooxygenase-2 (COX-2), monocyte chemoattractant protein-1 (MCP-1), and some others - to the mediating mechanisms of damage to large and medium cerebral arteries [86, 127–129] . For small arteries, due to the technical limitations of their imaging, the assessment of the lesion is carried out according to the data. This imposes serious limitations on the use of MRI in clinical practice as a recognition of early changes in the vessel wall. It is important to note that the quantitative study of the above biomarkers in predicting the risks of developing ICH remains debatable, despite their significant correlation with the severity of damage to the vascular wall of the cerebral arteries. The reason for this is the low specificity of the biomarkers, since most of these biomolecules are produced not only by the ECs or VSMCs, but also by leukocytes and platelets [163]. This requires the identification of a more specific biomarker associated with damage to the vascular elements of the cerebral arteries, in addition, these biomarkers do not reflect cellular function. There is already ample evidence that miRNAs play an important role in the processes underlying the normal function of ECs and VSMCs and their dysfunction [164–169]. And the fact that some miRNAs are found freely circulating in biological fluids indicates an early manifestation of damage to the vascular wall of cerebral arteries. Due to their specificity and ability to be detected in the early stages of disease development, circulating miRNAs can undoubtedly be considered as predictors of the onset of ICH (Fig. 6).

### Circulating miRNAs as biomarkers of neuroinflammation

As discussed above, neuroinflammation after ICH is a complex process of interaction between the immune and nervous systems, which occurs in response to damage to the nervous tissue from the hematoma mass effect and can continue for a long period after the hemorrhage [86]. In addition, the lysis of erythrocytes and the reaction of the brain tissue to decay products leads to the death of neurons, in response to which the process of neuroinflammation is triggered as a neuroprotective mechanism that provides protection for intact tissue: microglia are activated, infiltration by granulocytes and lymphocytes of the brain tissue, secretion by immunocompetent and glial pro- and anti-inflammatory cytokines, chemokines, and ROS cells [86, 99]. Adverse consequences and complications of ICH are associated with secondary damage and the development of neuroinflammation. Therefore, recognizing and determining the extent of the inflammatory process at an early stage is essential for optimizing immune-targeted treatment. On the other hand, diagnostic difficulties are due to nonspecific changes according to neuroimaging data. It has been repeatedly shown that miRNAs play a direct role as pro- or anti-inflammatory regulators after ICH, their presence in biological fluids presents the possibility of their use as prognostic biomarkers for the development of neuroinflammation. Hematoma growth and a large hematoma volume are associated with an unfavorable course of the disease, including the activation of inflammatory cascades. To date, there are already successful results from several studies that have shown that circulating miRNAs can be used to predict hematoma growth after ICH [59, 147]. In addition, besides EVs or protein complexes, miRNAs are expressed in erythrocytes, and due to their lysis, circulating miRNAs in apoptotic bodies can enter the bloodstream or CSF (when blood enters the lateral ventricles) and can potentially provide easy and rapid testing in clinical populations, helping to predict and monitor the degree of development of neuroinflammation [142, 143].

Prolonged activation of microglia or accumulation of inflammatory cytokines may interfere with recovery of brain function after injury, disrupt oligodendrocyte maturation, limit neuronal regeneration, and impair synapse formation. The results of experimental and clinical studies show that after ICH, the sensitivity of the patient's brain to further injuries increases. These processes and their consequences are defined as tertiary brain damage. Combined with acute and secondary cellular reactions such as cell death and metabolic disturbances, the tertiary injury phase may persist for months after hemorrhage [170, 171]. These active processes can be a goal for predicting/monitoring the condition of patients by determining changes in the expression level of circulating miRNAs for therapy correction. But this requires further study with prolonged (up to 6 months) follow-up of patients who have undergone ICH.

### Limitations in use of circulating miRNAs as biomarker in ICH

There are several classifications of biomarkers, the main ones: by type of molecule (for example, miRNA) and by task. Depending on the task, the following groups of biomarkers are distinguished: 1) diagnostic, which make it possible to establish the presence or absence of a disease and its severity; 2) prognostic, allowing to assess the risk of developing the disease or its complications; and 3) therapeutic, which help evaluate or predict the effectiveness of drug therapy [172, 173]. It is important to note that not every laboratory indicator can be called a biomarker. The main thing that distinguishes a biomarker is its statistically substantiated relationship with the disease, complication, and treatment effect, which is expressed in the following characteristics: statistical significance of differences in the selected characteristic, receiver operating characteristic curve (ROC), Kaplan–Meier curves, threshold value with calculated sensitivity and specificity, predictive value of the biomarker, likelihood ratio for the presence and absence of the trait and accuracy index [172, 173]. In most studies, circulating miRNAs are named as potential biomarkers based on their statistically significant increase or decrease in expression in ICH [41, 43–45, 49, 58, 59, 147–160]. To search for biomarkers, two main approaches are used: hypothesis-based and discovery-based. In the first approach, the researcher assumes that this indicator may be a biomarker of a certain disease since he knows the pathogenesis of this disease and the possible role of the indicator in it. The second approach screens a large number of molecules that may be associated with the disease. In the case of circulating miRNAs and ICH, there is a first approach [37, 174]. If we summarize all the requirements for a biomarker, we can highlight the following: it must have high sensitivity and specificity, be measured using reliable test systems, be determined in easily accessible biomaterial, be easily measured in real time, have an established reference interval and a proven relationship with disease or efficacy treatment. These requirements are the reason for the low rate of translation of thousands of potential biomarkers into clinical practice, and circulating miRNAs in ICH are no exception. Thus, among the main limitations of the clinical use of circulating miRNAs in the diagnosis and prognosis of ICH are: Firstly, the design and methodology of studies in this area are very heterogeneous, many studies are limited by a small sample size, some of them are retrospective. Secondly, due to the high heterogeneity of study designs, applied threshold values to determine changes in the expression profile of circulating miRNAs and the statistical processing methods used, the efficiency and quality of meta-analyses decreases. Thirdly, circulating miRNAs in ICH have limitations for clinical practice due to their low specificity. Their concentrations may largely depend on the use of anticoagulants, previous thrombotic complications, a concurrent systemic proinflammatory response, pregnancy, and other factors. There are a variety of pathogenetic subtypes of hemorrhagic stroke (hemorrhagic transformation of IS, subarachnoid hemorrhage (SAH) due to rupture of IAs, traumatic hemorrhages, hemorrhages due to genetic diseases, etc.). Therefore, the clinical application of circulating miRNAs requires identifying situations in which they should not be used for prediction. Fourthly, epidemiological (ethnic factor) and climatic-geographical factors (cold climate). And in lastly, discrepancy between the concentration of circulating miRNAs and the volume and severity of ICH due to the presence of the BBB: it is noted that even with impaired barrier permeability, the resulting hypoperfusion can prevent the penetration of circulating miRNAs into the bloodstream. The most promising is the use of circulating miRNAs for preliminary assessment of damage to the vascular wall and identification of individual subgroups of strokes. Also, assessing changes in the level of circulating miRNAs over time against the background of an increase in the volume of the hematoma may indicate the progression of the disease. Integration of circulating miRNAs into a clinical decision-making algorithm can improve the quality of patient management by more accurately identifying subgroups with an unfavorable prognosis, personalizing therapy when predicting response to it, and calculating the likelihood of disease progression or recurrence when assessed over time. However, prospective clinical and interventional studies are required before implementation into clinical practice.

In general, based on previous studies, we can conclude that any differential information about the volume, location, timing, and type of stroke is difficult to prove based solely on the concentration of circulating miRNAs in biological fluids. That is why the main role of circulating miRNAs in ICH at the moment should be considered to be a complement to existing standard diagnostic methods. The authors of most original studies, meta-analyses, and systematic reviews in the study of stroke agree that single circulating miRNAs do not have levels of sensitivity and specificity sufficient for clinical use, so the development of multimarker panels is becoming increasingly relevant.

**Fig. 7:**
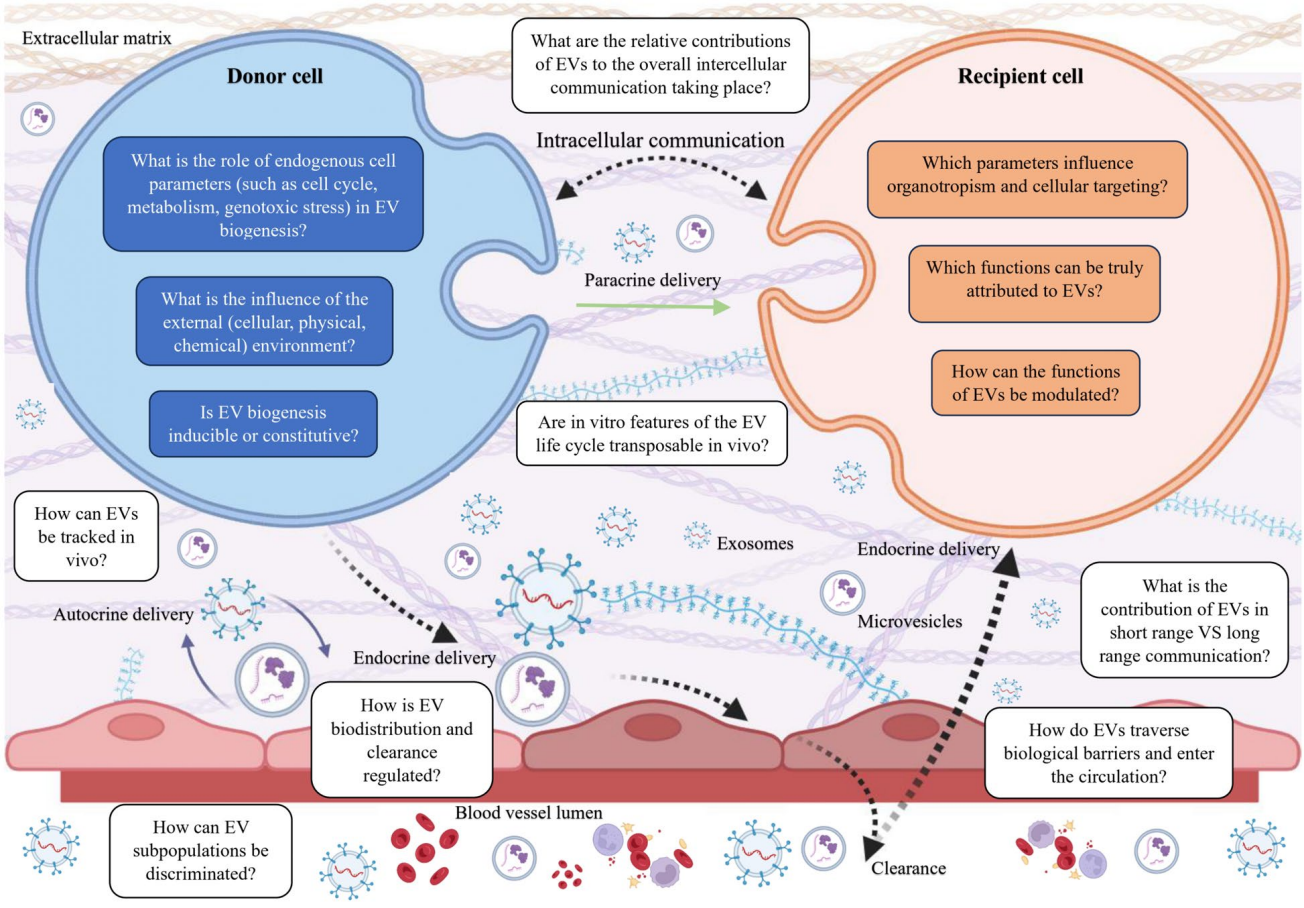
Limitations of the clinical use of microRNAs (miRNAs) in extracellular vesicles (EVs) as non-invasive biomarkers. Future challenges: 1) EVs biogenesis, 2) dynamics in extracellular, 3) uptake and functional delivery of EVs cargo, and 4) global role of EVs in intercellular communication

The significance of miRNAs in EVs as biomarkers of cerebrovascular diseases has been widely studied. To date, there has been significant progress in understanding the biological characteristics and functions of EVs in physiological and pathological conditions. As natural nanocarriers of miRNA of parent cells, EVs are effective mediators of intertissue communication in such a complex disease as ICH. In addition, preclinical and clinical studies demonstrate that miRNAs in EVs have great potential to serve as noninvasive biomarkers of CVDs; however, they are far from clinical application due to several obstacles, such as the significant heterogeneity of EVs populations or the lack of uniform quality control criteria (Fig. 7) [175–177]. Advancement of fundamental research in the field of studying miRNAs in the composition of EM will contribute to their wider use in the diagnosis and prediction of ICH in the future.

One way or another, any stroke biomarker being studied, including circulating miRNAs, should provide answers to priority clinical questions: whether the patient will or has had a stroke; whether the stroke is IS or ICH; is there a high risk of hemorrhagic transformation of IS; whether the patient has sufficient rehabilitation potential to carry out personalized rehabilitation treatment.

## Identification of candidate miRNAs as therapeutic and non-invasive diagnostic tools

**Fig. 8:**
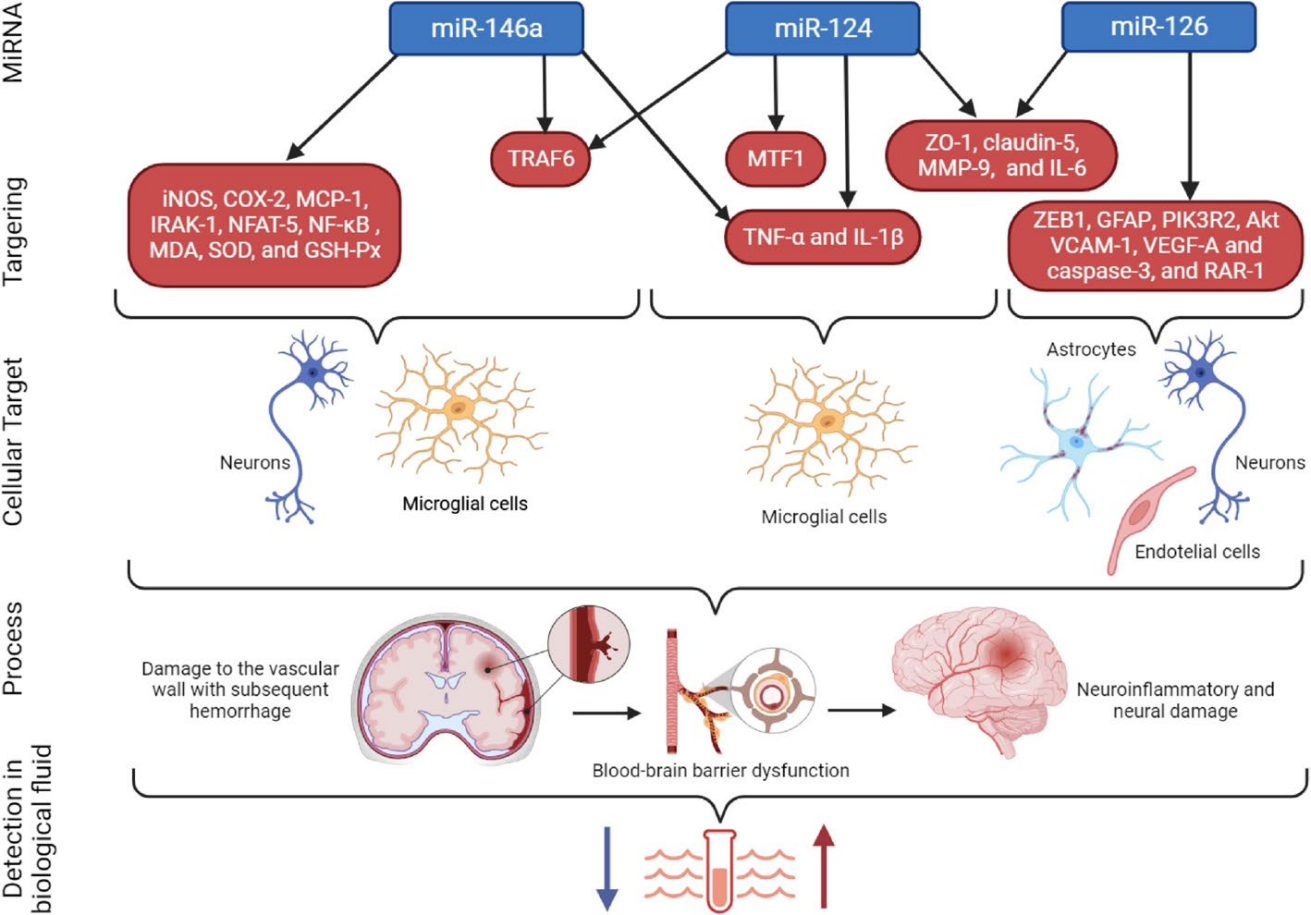
Schematic illustration of the most common microRNAs (miRNAs) studied in non-traumatic intracerebral hemorrhage (ICH) as therapeutic targets and non-invasive biomarkers. Notes: iNOS, Nitric oxide synthase; COX-2, Cyclooxygenase-2; MCP-1, Monocyte chemoattractant protein-1; IRAK1, Interleukin-1 receptor-associated kinase1; NFAT5, Nuclear factor of activated T cells 5; IL-6, Interleukin 6; TNF-a, Tumor necrosis factor alpha; IL‐1β, Interleukin 1 Beta; ZO-1, Zonula occludens-1; RAR-1, Protease activated receptor-1; TGF‑β1, Transforming growth factor β1; MDA, Malondialdehyde; SOD, Superoxide dismutase; GSH-Px, Glutathione peroxidase; TNF-α, Tumour necrosis factor alpha; MMP-9, Matrix metalloproteinase 9; PI3K, Phosphoinositide 3-kinases; Akt, RAC-alpha serine/threonine-protein kinase; VCAM-1, Vascular cell adhesion molecule 1; PIK3R2, Phosphatidylinositol 3-kinase regulatory subunit beta; TRAF6, TNF receptor associated factor 6; NF-κB, Nuclear factor kappa-light-chain-enhancer of activated B cells; ZEB1, Zinc finger E-box binding homeobox 1; GFAP, Glial fibrillary acidic protein; MTF1, Metal regulatory transcription factor 1; VEGF-A, Vascular endothelial growth factor A

In this review, we identified miR-146a, miR-124 and miR-126 reported by more than one study, and these miRNAs had different expression profiles in ICH models of *in vitro* and *in vivo* studies; moreover, they have been detected in the biological fluids of patients with ICH (Fig. 8) [37–40, 44, 46, 50, 58–60, 69, 70, 147, 151–153, 158, 160]. However, a more in-depth study of these miRNAs is fundamental to expanding our knowledge of the role these miRNAs play in ICH.

### Selection of miR-146a

It has been established that miR-146a is a well-expressed miRNA in various types of mammalian cells and has numerous evidence of its participation in inflammation, differentiation, and the functioning of specialized cells of the adaptive and innate immunity [178]. From available data it is known that miR-146a plays a significant role in neuroinflammation in various diseases of the CNS, such as IS and TBI, Alzheimer's disease, Parkinson's disease, Multiple Sclerosis, Prion diseases, Japanese encephalitis, and Herpes encephalitis [178–181]. In addition, some researchers demonstrate the role of this miRNA in neuroinflammation in ICH [50, 60]. In particular, the above results indicate that miR-146a-induced reduction in the expression of several target genes, including interleukin-1 receptor-associated kinase 1 (IRAK-1), nuclear factor of activated T-cells 5 (NFAT-5), and tumor necrosis factor receptor associated factor 6 (TRAF6)/NF-κB, has neuroprotective properties, as well as functional improvements in rats after ICH due to reducing neuronal apoptosis and neuroinflammation [50, 60]. NF-κB has been shown to play a central role in CVDs. NF-κB plays a good role in the development, inhibition and/or activation, and proliferation of lymphocytes, as well as in the regulation of proinflammatory and anti-inflammatory functions of dendritic cells and macrophages [182]. It was found that brain ECs transcripts regulated by NF-κB may include not only miR-146a, but also proinflammatory cytokines, chemokines, cell adhesion molecules, and nitric oxide synthase (NOS) [183, 184]. It has been demonstrated that miR-146a negatively modulates NF-κB through downregulating IRAK-1 and TRAF-6 in brain ECs [185, 186]. Have evidence that proinflammatory cytokines (e.g., TNF-α, IL-1, and IL-6), and chemokines (e.g., CC-chemokine ligand 2 (CCL2), CC-chemokine ligand 5 (CCL5), and chemokines chemokine (C-X-C motif) ligand 8 (CXCL8)) are transcriptional targets of NFAT5 in stroke [187]. Furthermore, there appears to be crosstalk between NFAT-5, NF-κB and miR-146a, where the latter is important for BBB development. In addition, separate experiments have shown that circulating miR-146a can be recognized in whole blood and serum, and since the expression level of this circulating miRNA is significantly altered in patients with VNK compared to healthy individuals, it is a priori hypothesized that this miRNA-146a may to be a promising diagnostic biomarker in the near future.

### Selection of miR-124

MiR-124 is an essential regulator of the developing brain and is also structured in mature neurons of the adult brain. MiR-124 has important functions in the CNS. These functions correlate with its involvement in neurotransmission, modulating synaptic morphology, and neuronal development [188, 189]. Dysregulation of miR-124 is associated with the development of CVDs such as ICH. Recently, abnormal expression of miR-124 was shown to be associated with the occurrence and development of ICH via regulating microglia function [58, 69, 70]. For instance, Fang et al., speculated an association between miR-124-3p with microglial injury in ICH via regulate TRAF6 in vitro [58]. In particular, it was demonstrated that miR-124-3p was targeted TRAF6 to regulate the NLRP3 expression, thereby modulating the secondary inflammation of microglia after ICH. Additionally, the expression levels of brain-specific miR-124 in the serum of ICH patients was downregulated. Important find in this study was a negative correlation has been identified between miR-124-3p expression and the Modified Rankin Score (mRS) score in ICH patients. Wang et al., identified that MTF1 as a predicted target of miR-124-3p, and enrichment of miR-124-3p target gene in microglia activation [69]. Other study was demonstrated that miR-124-containing exosomes significantly attenuates brain injury in a rodent model of bacterial collagenase-induced ICH. Specifically, intranasal delivery of miR-124-containing exosomes attenuates neurological deficits, brain edema, BBB leakage and apoptosis [70]. In addition, miR-124-containing exosomes reduced the number of brain-infiltrating immune cells, reducing the levels of inflammatory cytokines in the brain after ICH.

Among circulating miRNAs, circulating miR-124 is also the most frequently mentioned as a biomarker of ICH [58, 151, 152, 160]. For instance, to differentiate ICH and IS, miR-124-3p and miR-16 were studied: it was noted that the highest level of miR-124-3p in the plasma of patients with ICH occurs in the first 6 hours after the onset of symptoms of the disease with its subsequent decrease. On the contrary, the highest concentrations of miR-16 were observed in patients with IS admitted later from the onset of the disease (6–24 hours), compared to patients with ICH. Thus, miR-124-3p may be potential differential marker of IS and ICH during the acute period of the disease. However, given that miR-124-3p also enters the bloodstream during IS, the question of its reliability for differentiating ICH and IS remains open [190–192]. Existing studies have altered the expression profile of miR-124-3p in ICH and IS, and this finding suggests that the detection of circulating brain-specific miR-124-3p may be a consequence of direct brain tissue injury.

### Selection of miR-126

During the last years miR-126 have emerged as key regulator of several physiological and pathophysiological processes in the vascular wall. MiR-126 is an endothelial cell-specific miRNA that is known to be involved in angiogenesis [193]. MiR-126 controls the functions of cerebral vascular ECs under hypoxic conditions, while enhancing post-IS angiogenesis and neurogenesis [194]. MiR-126 alleviates BBB dysfunction by preventing the increase in MMPs and stopping the decrease in junctional proteins, including the levels of claudin-5, ZO-1, and occludin [195]. Moreover, miR-126 is involved in vascular inflammation and neuroinflammation [195, 196]. Amongst miRNAs, miR-126 is one of the most highly-expressed and highly-studied in ICH [37–40, 44, 46]. Functionally, miR-126 has multiple functions. In ICH, upregulation of miR-126 reduces ECs and neuronal death, where VEGF-A, caspase-3, ZEB1, GFAP, etc., are targets. Moreover, miR-126 reduces brain edema and BBB permeability, inhibits immune cells infiltration to brain, and microglial activation. Due to these important functional activities, miR-126 has become a target for therapeutic intervention in ICH. In addition, was identified that circulating miR-126 that were correlated with the neurological deficit severity (National Institutes of Health Stroke Scale (NIHSS) scores) and clinical outcomes (mRS scores at 90 days) of ICH severity and circulating miR-126 expression levels positively correlated with perihematomal edema (PHE), providing prognosis information on recovery [44, 147]. Considering the significance and complexity of miR-126 and its regulatory network in strokes, it is essential to further investigate the role of miR-126 in the initiation and progression of ICH to identify potential clinical applications.

## Conclusions and outlook

**Fig. 9:**
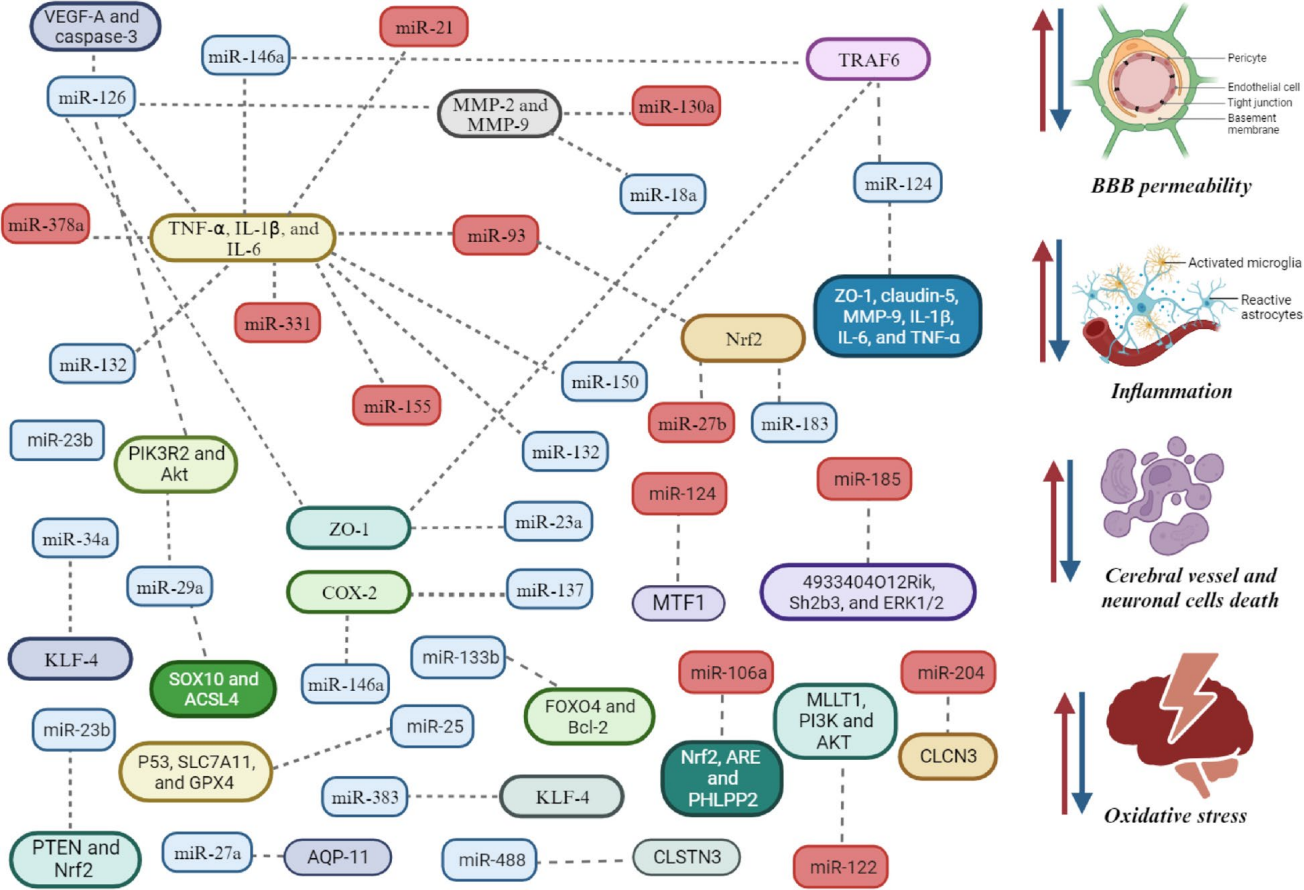
Demonstration of cross-regulation moments of common target genes for certain microRNAs (miRNAs) during non-traumatic intracerebral hemorrhage (ICH). MiRNAs highlighted in blue demonstrate a positive therapeutic effect on the main pathogenetic links of ICH as a decrease in the permeability of the blood-brain barrier (BBB) and edema, inflammation, generation of reactive oxygen species (ROS) and apoptosis); miRNAs that are highlighted in red show the opposite effect, as an increase in the permeability of the BBB and edema, inflammation, generation of ROS and apoptosis. Notes: PIK3R2, Phosphatidylinositol 3-kinase regulatory subunit beta; IL-6, Interleukin 6; TNF-a, Tumor necrosis factor alpha; IL‐1β, Interleukin 1 Beta; MMP-2, Matrix metalloproteinase 2; MMP-9, Matrix metalloproteinase 9; COX-2, Cyclooxygenase-2; ZO-1, Zonula occludens-1; Nrf2, Nuclear factor erythroid 2–related factor 2; KLF-4, Krüppel-like factor 4; TRAF6, TNF receptor associated factor 6; P53, tumor protein p53; SLC7A11, Cystine/glutamate transporter; GPX4, Glutathione peroxidase 4; FOXO4, Forkhead box O4; Bcl-2, B-cell leukemia/lymphoma 2; 4933404O12Rik, RIKEN cDNA 4933404O12; Sh2b3, SH2B adaptor protein 3; ERK1/2, Extracellular signal-regulated kinase 1/2; CLSTN3, Calsyntenin 3; SOX10, SRY-box transcription factor 10; ACSL4, Acyl-CoA synthetase long chain family member 4; MTF1, Metal regulatory transcription factor 1; ARE, Antioxidant responsive element; PHLPP2, PH domain and leucine rich repeat protein phosphatase 2; MLLT1, Myeloid/lymphoid or mixed lineage leukemia translocated to 1; PI3K, Phosphoinositide 3-kinases; Akt, RAC-alpha serine/threonine-protein kinase; CLCN3, Chloride voltage-gated channel 3

ICH is a huge medical, social, and economic problem and is now among the leading causes of severe and long-term disability. Despite recent progress, therapies that improve survival after ICH remain limited. Therefore, the molecular mechanisms of this complex pathogenetic process are being widely studied in search of new diagnostic and therapeutic possibilities. Based on current data, the important role of miRNAs as key mediators in the molecular processes underlying the pathogenesis of ICH becomes apparent. The identification of miRNAs and their respective target genes may offer new therapeutic strategies. However, as is already known, there may not be a one-to-one correspondence between a miRNA and its target mRNA, i.e., a miRNA may have several target mRNAs, and an mRNA may have several corresponding miRNAs (Fig. 9). All this creates difficulties in the potential application of miRNAs in clinical practice, requiring further research at the preclinical level.

The search for and development of effective and sensitive diagnostic and prognostic biomarkers in ICH is currently an important scientific area in neurology/neurosurgery. The study of circulating miRNAs, with the help of which the possibility of predicting complications and adverse outcomes of ICH, remains an urgent task, since it will allow individualizing the approach to the treatment and rehabilitation of patients. Of relevance are studies devoted to revealing the mechanisms of the influence of miRNAs on risk factors (chronic hypertension and atherosclerosis) of ICH, revealing not only therapeutic possibilities, but also the identification of circulating miRNAs as biomarkers of early damage to the vascular wall and the inflammatory process in order to predict the possibility of developing ICH. However, if we compare the number of studies carried out with IS, then the study with ICH is extremely small. At the same time, according to statistics, brain ischemia is more common, and hemorrhage in the brain tissue is considered a more severe variant of the course, which also requires a lot of attention from researchers. In addition, as can be seen from Table 5, circulating miRNAs are associated with a higher incidence of complications or adverse outcomes of ICH. Such an association is not enough to classify circulating as diagnostic or prognostic biomarkers; an in-depth statistical analysis is required, which will allow to identify multifactorial relationships, evaluate the significance, and calculate the diagnostic or prognostic characteristics of the test with the obligatory use of ROC analysis. It is also necessary to increase the number of studies on the use of circulating miRNAs in the differential diagnosis of stroke subtypes (ICH vs SAH or IS). Nevertheless, further study of circulating miRNAs makes it possible to predict the level of damage to the cerebral vessel wall and the likelihood of its rupture, as well as to confirm the fact of an event that occurred in real time and determine the period of ICH (acute, subacute and chronic), distinguish its subtypes and even judge the prognosis.

## Data Availability

The raw data supporting the conclusions of this article will be made available by the authors, without undue reservation.
